# Interface States in Space-Time Photonic Crystals: Topological Origin, Propagation, and Amplification

**DOI:** 10.1021/acsphotonics.5c02806

**Published:** 2026-05-21

**Authors:** Alejandro Caballero, Thomas F. Allard, Paloma A. Huidobro

**Affiliations:** † Departamento de Física Teórica de la Materia Condensada, 16722 Universidad Autónoma de Madrid, E28049 Madrid, Spain; ‡ Condensed Matter Physics Center (IFIMAC), 16722 Universidad Autónoma de Madrid, E28049 Madrid, Spain; ∥ Instituto Nicolás Cabrera (INC), 16722 Universidad Autónoma de Madrid, E28049 Madrid, Spain

**Keywords:** time-varying media, topological photonics, space-time photonic crystals, interface states

## Abstract

Studying the topology of spatiotemporal media poses a fundamental challenge: their remarkable properties stem from breaking spatial and temporal symmetries, yet this same breaking obscures their topological characterization. Here, we show that space-time symmetries persist in crystals with traveling-wave modulations whose velocities can be either lower (subluminal) or higher (superluminal) than the speed of light, enabling the study of their topological properties and the prediction of spatiotemporal interface states. For each modulation regime, we use a Lorentz transformation to a frame in which the modulation depends on only one of the transformed variables. Then, we identify a conserved joint parity-time-reversal symmetry in the new variables that enforces the quantization of a spatiotemporal Zak phase, elevating it to a $Z_{2}$ topological invariant. Finally, we calculate the associated interface states and uncover unique features arising from time-varying effects, including selective directional amplification, propagation along subluminal and superluminal boundaries, frequency- and momentum-converted replicas, and broadband amplification even in the absence of momentum gaps. Our framework holds for spatiotemporal modulations of any velocity, providing a unified description that encompasses photonic time crystals and clarifies their topological origin.

## Graphical abstract

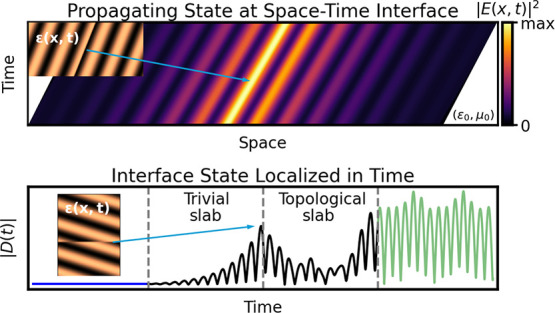

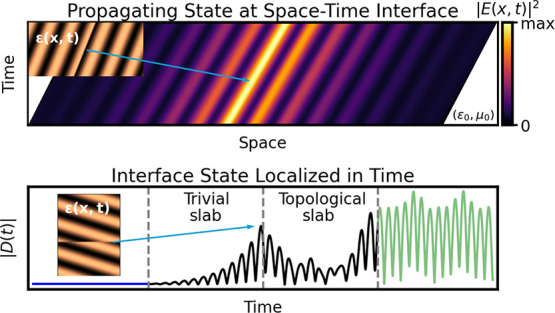

## Introduction

In time-varying media, the temporal modulation of a material’s properties lifts the usual constrains of passive systems by breaking fundamental symmetries such as continuous time translation.^1,2^ As a result, energy conservation no longer holds, enabling light amplification and frequency conversion. When temporal and spatial modulations are combined, additional constrains are lifted, and further wave manipulation can be achieved through frequency-momentum transitions.^3–5^ In particular, periodic space-time modulations of the traveling-waveform *f*(*x*, *t*) = *f*(*x* – *c*_g_ *t*), where *c*_g_ corresponds to the modulation speed, have attracted significant attention since early studies.^6–8^ Specifically, since the material itself is stationary, the grating speed can take any value up to infinity while respecting special relativity. As a result, two qualitatively distinct regimes emerge, determined by the modulation speed relative to the speed of light in the medium: a subluminal (space-like) regime characterized by conventional band gaps where momentum becomes a complex magnitude, and a superluminal (time-like) regime featuring momentum gaps where frequency is complex, known as *k*-gaps.^8,9^ Furthermore, such modulations introduce a linear-momentum bias that enables magnet-free nonreciprocity, manifested as asymmetric band gaps supporting unidirectional propagation,^10–14^ as well as synthetic Fresnel drag effects in the long-wavelength limit.^15^ Interestingly, such spatiotemporal (ST) phenomena originate fundamentally from wave interference effects, allowing their experimental realization across diverse platforms: from mechanical waves in water,^16,17^ elastic^18,19^ and acoustic systems,^20–22^ to electromagnetic (EM) waves spanning a wide range of frequencies in metasurfaces,^23^ transmission lines^24,25^ and ENZ materials.^26,27^

Symmetries also play a central role in topological physics, where the Altland–Zirnbauer classification groups topological insulators into ten classes according to the presence or absence of three key symmetries: time-reversal, particle-hole, and chiral.^28,29^ Depending on the dimensionality of the system, each class may host topological phases identified by a characteristic bulk topological invariant. A quantized invariant predicts the emergence of robust boundary states between materials of different phases, a principle known as bulk-boundary correspondence. These concepts can be applied to photonic crystals (PhCs), as topological invariants can also be attributed to their band structures.^30–33^ However, PhCs typically lack chiral and particle-hole symmetries, which, together with the modified parity of the time-reversal operator $\left(T^{2}=+1\right)$ due to their bosonic nature, it limits the number of possible nontrivial phases. Crucially, richer topological effects can emerge by invoking additional symmetries such as parity-time-duality,^34,35^ which restores a fermionic-like time-reversal operator and enables the photonic analogue of the quantum spin Hall effect in photonics.^36,37^ Moreover, crystalline symmetries reveal finer topological phases within classes that would otherwise appear trivial.^38–42^ For instance, one-dimensional (1D) PhCs present topological states thanks to the presence of parity (inversion) symmetry,^43^ with their topological invariant being the Zak phase.^44^

Recently, it has been shown that the band structures of photonic time crystals (PTCs), where the optical properties of a homogeneous material periodically vary in time instead of space as in PhCs, are also classified by a quantized Zak phase when the modulation preserves temporal inversion symmetry. In turn, interface states localized in time emerge,^45^ which can be robust against disorder for chiral-symmetric modulations.^46^ Localization in time has also been proposed in different time-varying systems, as in experiments with Floquet lattices,^47,48^ or two-level systems with parity-time symmetry.^49^

Beyond purely temporal modulations, combining space and time variations produces ST systems with a remarkably rich topological landscape. Two broad regimes can be distinguished. First, in decoupled modulations of the form *f*(*x*, *t*) = *g*(*x*)*h*(*t*), space and time act as independent degrees of freedom, enabling higher-dimensional topological phases such as D+1 Floquet insulators,^50,51^ synthetic dimensions in frequency space,^52–54^ and systems with both momentum and frequency band gaps that allow for localization in space and time.^55^ Second, in coupled ST modulations, exemplified by traveling-wave type modulations, time is no longer independent, and the system remains effectively 1D, which forbids the well-established Chern classification applicable to decoupled modulations, although a recent extension has been proposed through a synthetic vector potential approach.^56^ Previous studies in acoustic systems have investigated the topology of subluminal modulations in experiment by invoking an adiabatic approximation, which leads to a decoupled 1 + 1D formalism with its associated Chern number, but overlooks the effects introduced by the dynamical modulation.^57^ Other works, avoiding this approximation, revealed interface states accompanied by frequency-converted replicas at frequencies far from the band gap.^58^ However, in that case, the topological characterization relied on a time-dependent Zak phase, leaving open the question of whether a symmetry-protected invariant underlies the existence of these states. Furthermore, the extension of any topological characterization into the superluminal regime has so far remained unexplored in the literature.

In this work, we answer this question affirmatively by considering a PhC whose permittivity is modulated in a traveling-waveform and analyzing the ST symmetries it supports. First, we develop the formalism for subluminal modulations, whose spatial nature enables a direct comparison with well-established spatial PhCs, and then extend it to the superluminal regime. For both types of modulations, we perform a Lorentz transformation to a frame in which the modulation depends on only one of the transformed variables, allowing us to identify the conserved symmetries and to show that a combined parity-time-reversal transformation remains invariant. This symmetry enforces the quantization of a spatiotemporal Zak phase defined along the Brillouin Zone (BZ) in the new frame, thereby establishing a $Z_{2}$ topological invariant. Furthermore, by calculating the band energy density, we distinguish the two resulting phases given by such an invariant as a trivial phase and an obstructed atomic limit. This allows us to predict and unveil the interface states that arise between slabs of different ST Zak phases, considering different types of boundaries depending on the modulation regime, including spatial, temporal, and spatiotemporal ones. Importantly, these states persist regardless of the modulation speed, and they are pinned at the bulk band-crossing position, confirming their topological origin. Owing to their ST nature, the interface states present unique features such as selective directional amplification, frequency- and momentum-converted replicas, as well as propagation along subluminal and superluminal boundaries, and broadband amplification even in the absence of momentum gaps. Finally, we analyze the effect of relevant perturbations on the modulation, demonstrating the robustness of the interface states and clarifying the conditions under which they remain topological.

## Methods

### Bloch–Floquet Theory of Traveling-Wave PhCs

We consider a medium whose permittivity is modulated in space and time following the traveling form

$$\epsilon (x,t)=\epsilon_{0}\epsilon_{m}[1+\alpha \cos(gx-\Omega t)]$$ 1

with ϵ_*m*_ the background relative permittivity of the medium, *g* and Ω the spatial and temporal modulation frequencies with periods *a* = 2π/*g* and *T* = 2π/Ω, and α the modulation strength. The permeability of the medium μ = μ_0_μ_m_ is considered as constant, where μ_*m*_ is the background permeability, and $c_{0}=1/\sqrt{\epsilon_{0}\mu_{0}}$ is the speed of light in vacuum. As sketched in Figure 1a, this specific modulation, where space and time are coupled, creates a moving grating with phase velocity *c*_g_ = Ω/*g* along the *x* direction. Since the material itself is not moving, this grating speed can take any value up to infinity while respecting special relativity. This allows for the same model described by eq 1 to host different regimes depending on whether the grating velocity is lower (subluminal) or higher (superluminal) than the speed of light in the medium $c=c_{0}/\sqrt{\epsilon_{m}\mu_{m}}$ ,^15,59^ leading to drastically different behaviors. First, we focus on the subluminal regime, defined for our traveling-wave modulation as $0\leq c_{g}\leq c/\sqrt{1+\alpha }$ ,^59^ with the upper bound marking the onset of the luminal regime, which we will not consider here due to the absence of the notion of band structure. In a later section, we study the superluminal regime by considering modulation velocities ranging in $c/\sqrt{1-\alpha }\leq c_{g}\leq \infty$ .

**Figure 1:**
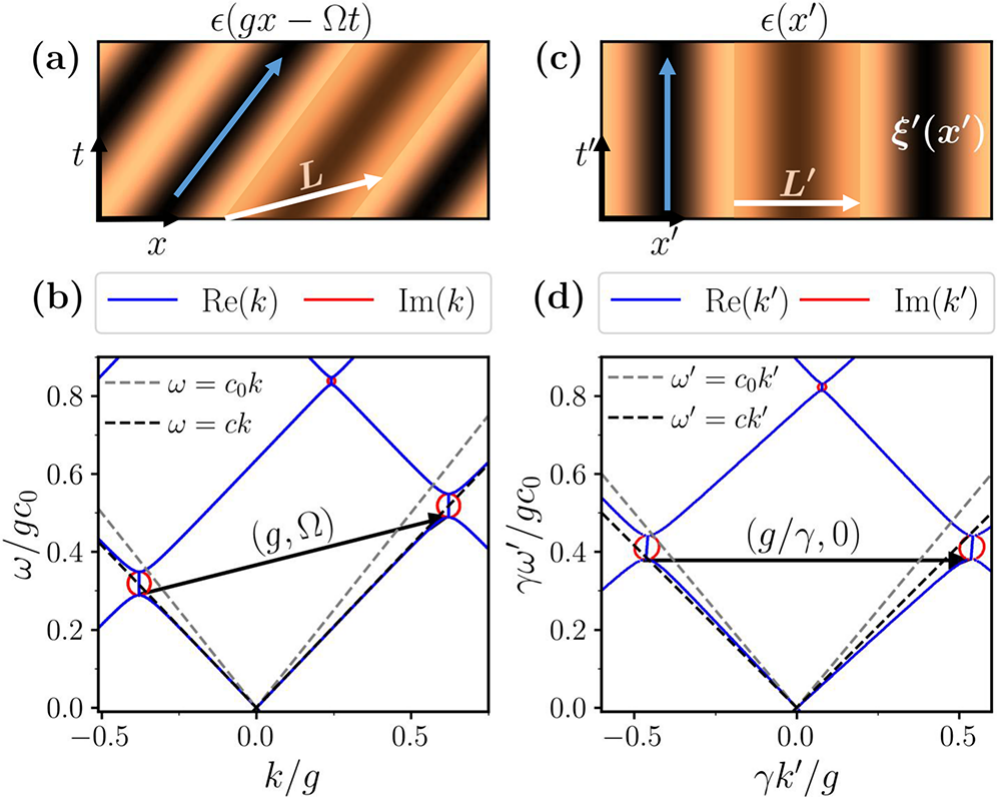
Spatiotemporal photonic crystal in the laboratory and comoving frames for subluminal modulation. (a) Traveling-wave modulated permittivity in the lab-frame. A blue arrow signals the direction of the continuous space-time translation symmetry that defines the unit cell highlighted in the orange shaded region. The white arrow corresponds to the lattice vector. (b) Band structure of a STPhC for a modulation speed *c*_g_ = Ω/*g* = 0.2*c*_0_, modulation strength α = 0.3 and ϵ_*m*_ = μ_*m*_ = 1.2. Gray and black dashed lines correspond to the dispersion relation in free space and in the unmodulated material, respectively. Black arrow represents the reciprocal lattice vector **p**. (c) Modulated permittivity in the comoving frame. The previous magnitudes are now spatial-like, but a new magneto-electric coupling ξ*′*(*x′*) appears. (d) Band structure of the same STPhC in the comoving frame, with the black arrow now representing the transformed reciprocal vector **p** *′*.

We calculate the band structure of the spatiotemporal PhC (STPhC) by solving Maxwell’s equations, ∇ × **E** = –*∂*_*t*_ **B** and ∇ × **H** = *∂*_*t*_ **D**. Considering s-polarization and normal incidence, the latter equations can be written in matrix form as

$$\left(\begin{array}{ll} 0 & \partial_{x}\\ \partial_{x} & 0 \end{array}\right)\left[\begin{array}{l} E_{z}\\ H_{y} \end{array}\right]=\partial_{t}\left(\mathrm{M̂}(x,t)\left[\begin{array}{l} E_{z}\\ H_{y} \end{array}\right]\right)$$ 2

where **M̂**(*x*, *t*) corresponds to the constitutive matrix of the relevant components of the EM field

$$\mathrm{M̂}(x,t)=\left(\begin{array}{ll} \epsilon (x,t) & 0\\ 0 & \mu \end{array}\right)$$ 3

such that

$$\left[\begin{array}{l} D_{z}\\ B_{y} \end{array}\right]=\mathrm{M̂}(x,t)\left[\begin{array}{l} E_{z}\\ H_{y} \end{array}\right]$$ 4

Then, we use the Bloch–Floquet ansatz

$$\left[\begin{array}{l} E_{z}(x,t)\\ H_{y}(x,t) \end{array}\right]=e^{i(kx-\omega t)}\sum_{n}\left[\begin{array}{l} E_{n}\\ H_{n} \end{array}\right]e^{in(gx-\Omega t)}$$ 5

to derive an eigenvalue problem for the Bloch–Floquet amplitudes *E*_n_ and *H*_n_. This allows us to compute the dispersion relation as *k*(ω) for the subluminal regime, where the band gaps present complex momenta, and ω­(*k*) for the superluminal case, whose momentum gaps host complex frequencies. Finally, we also obtain the mode decomposition of the eigenfunctions of the STPhC (see the Supporting Information (SI) for detailed derivations).

We represent the obtained band diagram of the STPhC in the subluminal regime in Figure 1b, together with the dispersion relation in free space and in the unmodulated medium (dashed lines). In this regime, the band structure displays frequency band gaps akin to spatial PhCs, in contrast to the momentum band gaps present in PTCs^1^ or in the superluminal regime, as we will see in a later section. Interestingly, however, the gaps are asymmetric and appear at different frequencies for forward (*k* > 0) and backward (*k* < 0) waves, revealing the mechanism by which STPhCs enable one-way propagation.^3,4,12^

The asymmetric band structure stems from the coupling between spatial and temporal modulations induced by the traveling waveform of eq 1. Since the STPhC is effectively a 1D system, it is described by a single lattice vector. While the temporal modulation breaks continuous time translation symmetry, a traveling wave modulation exhibits a continuous ST translational symmetry that conserves a linear superposition of energy and momentum.^60^ This is highlighted by the blue arrow in Figure 1a. Such continuous symmetry of the system defines the unit cell, sketched as a shaded trapezoid, and a ST lattice vector **L** with both spatial and temporal components. The reciprocal lattice vector is also a ST one, **p** = (*g*, Ω), see Figure 1b, and its nonzero frequency component tilts the entire band structure and explains the asymmetry of the band gaps.

### Reference Frame Transformation

To facilitate the description of the crystal’s underlying ST symmetries, we reformulate our problem in a reference frame where the modulation depends on a single transformed variable, either space or time, depending on the modulation regime. We begin with the subluminal case, for which we choose a frame comoving with the modulation.^9,59,61–65^ To do so, we use Lorentz transformations to ensure that Maxwell’s equations are conserved, as we are interested in studying the different symmetries of the system. After a Lorentz boost in the *x* direction, the comoving coordinates read

$$x^{\prime }=\gamma (x-c_{g}t),y^{\prime }=y,z^{\prime }=z$$ 6

and

$$t^{\prime }=\gamma \left(t-\frac{c_{g}}{c_{0}^{2}}x\right)$$ 7

where the Lorentz factor $\gamma =1/\sqrt{1-(c_{g}/c_{0}{)}^{2}}$ . By transforming the EM fields accordingly,^66^ we then obtain Maxwell’s equations in the comoving frame

$$\left(\begin{array}{ll} 0 & \partial_{x^{\prime }}\\ \partial_{x^{\prime }} & 0 \end{array}\right)\left[\begin{array}{l} {E_{z}}^{\prime }\\ {H_{y}}^{\prime } \end{array}\right]={\mathrm{M̂}}^{\prime }(x^{\prime })\partial_{t^{\prime }}\left[\begin{array}{l} {E_{z}}^{\prime }\\ {H_{y}}^{\prime } \end{array}\right]$$ 8

with the transformed constitutive matrix

$${\mathrm{M̂}}^{\prime }(x^{\prime })=\left(\begin{array}{ll} {\epsilon^{\prime }}_{⊥}(x^{\prime }) & \xi^{\prime }(x^{\prime })\\ \xi^{\prime }(x^{\prime }) & {\mu^{\prime }}_{⊥}(x^{\prime }) \end{array}\right)$$ 9

whose elements in the new frame read

$${\epsilon^{\prime }}_{⊥}(x^{\prime })=\frac{\epsilon (x^{\prime })}{\gamma^{2}(1-\epsilon (x^{\prime })\mu c_{g}^{2})}$$ 10

$${\mu^{\prime }}_{⊥}(x^{\prime })=\frac{\mu }{\gamma^{2}(1-\epsilon (x^{\prime })\mu c_{g}^{2})}$$ 11

and

$$\xi^{\prime }(x^{\prime })=c_{g}\frac{\epsilon (x^{\prime })\mu -c_{0}^{-2}}{1-\epsilon (x^{\prime })\mu c_{g}^{2}}$$ 12

In the comoving frame, the constitutive parameters depend on the spatial coordinate *x′* only, and the modulation loses the explicit temporal dependence. More importantly, moving between frames leads to the material acquiring a bianisotropic coupling *ξ′*(*x′*) proportional to the grating velocity, resulting in a moving-medium type coupling between the electric and magnetic fields.^59^ This can be understood by noting that, in the lab frame, only the modulation propagates while the material remains stationary. Therefore, when changing to the moving frame, the modulation becomes static, but the material appears to be moving in the opposite direction, giving rise to the bianisotropic response *ξ′*(*x′*) akin to a moving medium.

We sketch the permittivity distribution in the comoving frame in Figure 1c. In this frame, there is a continuous ST translation symmetry along *t′*, such that the unit cell is defined with the spatial coordinate only as *x′* ∈ [−γ*a*/2, γ*a*/2], with lattice vector **L** *′* = (γ*a*, 0). Using the reciprocal coordinates in the comoving frame

$$k^{\prime }=\gamma \left(k-\frac{c_{g}}{c_{0}^{2}}\omega \right),{k^{\prime }}_{y}=k_{y},{k^{\prime }}_{z}=k_{z}$$ 13

and

$$\omega^{\prime }=\gamma (\omega -c_{g}k)$$ 14

we obtain the reciprocal lattice vector **p** *′* = (*g*/γ, 0) as well as the BZ defined as *k′* ∈ [−*g*/2γ, *g*/2γ]. Finally, transforming back to the laboratory frame, the corresponding lattice vector can be calculated as **L** = γ^2^ *a*(1, *c*_g_/*c*_0_^2^). This derivation highlights the importance of employing a Lorentz transformation rather than a Galilean one for our purposes, as the mixed nature of the space-time dimension cannot be taken into account without transforming the temporal variable.

With the lattice vector now established in the comoving frame, the periodicity of the crystal becomes explicit, namely, **M̂** *′*(*x′* + γ*a*) = **M̂** *′*(*x′*). This allows us to apply Bloch’s theorem to eq 8 and obtain the dispersion relation ω*′*(*k′*) together with the comoving-frame eigenfunctions

$${\left[\begin{array}{l} E^{\prime }(x^{\prime })\\ H^{\prime }(x^{\prime }) \end{array}\right]}_{k^{\prime },m}=e^{ik^{\prime }x^{\prime }}{\left[\begin{array}{l} {u^{\prime }}^{E}(x^{\prime })\\ {u^{\prime }}^{H}(x^{\prime }) \end{array}\right]}_{k^{\prime },m}$$ 15

where the subscripts of the electric and magnetic fields have been dropped for clarity, *u*_*k′*,*m*_^*E*/*H*^(*x′* + γ*a*) = *u*_*k*′,*m*_^*E*/*H*^(*x′*) correspond to the periodic part of the eigenfields, and *m* represents the band index (see SI).

Figure 1d shows the dispersion relation of the STPhC in the comoving frame. The absence of a frequency component in **p** *′* removes the tilted nature of the band structure observed in the laboratory frame [cf. Figure 1b], rendering the band gaps symmetric. However, the system still presents nonreciprocal features, as visible from the fact that ω′(−*k′*) ≠ ω′(*k′*). Indeed, an increased group velocity is observed for backward propagating waves, while forward propagating waves present a reduced one. This is a result of the nonzero magneto-electric coupling that appears in the comoving frame, which breaks time-reversal symmetry $T^{\prime }(t^{\prime }\to -t^{\prime })$ and, consequently, reciprocity.^67^

Therefore, although the reciprocal lattice vector is spatial-like in the comoving frame, the effective magneto-electric coupling induced by the frame transformation still underlies the breaking of fundamental symmetries as well as the emergence of nonreciprocal features. In the following, we analyze the impact of this bianisotropic coupling on the ST symmetries present in the comoving frame, as well as its consequences in the topological characterization.

## Results and Discussion

### Topological Characterization of STPhCs

In spatial nonmagnetic PhCs, only time-reversal T is present out of the three fundamental symmetries that define the tenfold way,^28,29^ which for general 1D systems classify them as trivial. However, crystalline symmetries can enrich PhCs with nontrivial topology.^39^ In 1D, this role is played by parity (inversion) symmetry P(x→−x) , which quantizes the Zak phase,^44^ defining it as a $Z_{2}$ topological invariant. This symmetry also establishes a direct connection between the parity of the Bloch functions and the existence of surface states,^43,68^ i.e., it establishes a bulk-interface correspondence.

Motivated by this context, we now discuss the topological characterization of a subluminal STPhC by studying the space–time counterpart of parity symmetry, defined in the comoving frame as $P^{\prime }\left(x^{\prime }\to -x^{\prime }\right)$ . Applying this transformation to eq 8 reveals that, although each entry of the material matrix is symmetric under $P^{\prime }$ , the presence of the magneto-electric coupling ξ*′* breaks the invariance of Maxwell’s equations. As a result, the direct generalization of the symmetry that enables nontrivial topology in static crystals is not possible, raising the question of whether STPhCs can still be topologically classified. Crucially, however, while $T^{\prime }$ and $P^{\prime }$ are individually broken, their combined operation $P^{\prime }T^{\prime }[(x^{\prime },t^{\prime })\to -\left(x^{\prime },t^{\prime }\right)]$ is preserved. Physically, $P^{\prime }T^{\prime }$ -symmetry has the same relevance as conventional PT -symmetry since both transformations are equivalent, as can be seen in eqs 6 and 7. In what follows, we examine whether this joint symmetry can play a similar role as parity symmetry does in 1D spatial PhCs.

To do so, we study the Zak phase defined with the eigenfunctions of eq 15 and along the comoving-frame BZ

$$\theta_{m}^{\mathrm{ST}}=i\int_{-g/2\gamma }^{+g/2\gamma }dk^{\prime }\langle {u^{\prime }}_{k^{\prime },m}|\partial_{k^{\prime }}{u^{\prime }}_{k^{\prime },m}\rangle_{\mathrm{M̂}\prime }$$ 16

where the integrand corresponds to the Berry connection and **u**′_*k*′,*m*_ = [*u*_*k*′,*m*_^*E*^, *u*_*k*′,*m*_^*H*^]^*T*^. Importantly, the Hermiticity of the constitutive matrix in eq 9, together with $P^{\prime }T^{\prime }$ symmetry, allows us to relate the left and right eigenvectors of the system (see SI).^69^ As a result, the conventional Berry connection can be used in place of the biorthogonal one, which would otherwise be required by the non-Hermitian nature of the differential operator in eq 8. This Berry connection is defined with a weighted scalar product over the unit cell given by

$$\left\langle \Psi_{1}^{\prime }|\Psi_{2}^{\prime }\rangle_{{\mathrm{M̂}}^{\prime }}=\int_{-\gamma a/2}^{+\gamma a/2}dx^{\prime }{\Psi_{1}^{\prime }}^{†}(x^{\prime }){\mathrm{M̂}}^{\prime }(x^{\prime })\Psi_{2}^{\prime }(x^{\prime }\right)$$ 17

The scalar product ensures the normalization of the comoving-frame eigenfunctions by establishing ⟨**Ψ** *′*_*k′*,*m*_|**Ψ** *′*_*k′*,*m*_⟩_**M̂** *′*_ = 1, with **Ψ** *′*_*k′*,*m*_ = [*E′*_*k′*,*m*_, *H′*_*k′*,*m*_]^*T*^.

Furthermore, building upon the results of ref 70 on the Hermitian Berry connection, one can show that $P^{\prime }T^{\prime }$ -symmetry alone allows the quantization of the ST Zak phase. The quantization defines θ_*m*_^ST^ as a $Z_{2}$ topological invariant, which means that one can distinguish between two different phases, delimited by a topological transition where the band gap must close. Therefore, solely from this property, we can predict the existence of interface states between two STPhCs with different topological phases.

To understand the physical meaning behind these two phases, we use eq 17 to establish a direct relation between Wannier centers and the ST Zak phase, as is done in the Hermitian case.^71^ We can then write

$${{\mathrm{x̅}}^{\prime }}_{m}=\langle {w^{\prime }}_{L^{\prime },m}|x^{\prime }|{w^{\prime }}_{L^{\prime },m}\rangle_{{\mathrm{M̂}}^{\prime }}=\gamma \theta_{m}^{\mathrm{ST}}/g$$ 18

with **w** *′*_*L′*,*m*_(*x′*) = γ*a*/2π∫d*k′*e^*ik^′^L^′^*^ **Ψ**′_*k′*,*m*_ the Wannier function associated with the eigenfields **Ψ**′_*k′*,*m*_ and *L*′ = *n*γ*a*, with n∈Z , labeling the position of the unit cell. Therefore, the quantization of θ_*m*_^ST^ enforced by $P^{\prime }T^{\prime }$ -symmetry translates directly into the quantization of the Wannier centers. In particular, since the Zak phase can only take the values 0, π (mod 2π), the Wannier functions can only be localized at the positions *x̅* *′* = 0, γ*a*/2, which correspond to the inversion centers of the unit cell.

In electronic insulators, Wannier centers mark the positions of electronic charge within the unit cell, effectively defining atomic orbitals.^71^ The quantization of *x̅*_*m*_^*′*^ thus distinguishes two phases, with orbitals localized at the cell center or edges. Within the band representation framework,^39,72,73^ these correspond in one dimension to trivial and obstructed (topological) phases, respectively. This formalism extends to PhCs, where EM energy density acts as an analogue of electronic charge,^74^ enabling the same physical interpretation of the two phases defined by the ST Zak phase in our photonic system. Indeed, this can be derived directly from the time-averaged energy density of the comoving-frame eigenfields. Integrating over the whole BZ, the latter density reads^66^

$$n_{m}(x^{\prime })=\int_{-g/2\gamma }^{+g/2\gamma }\frac{{E^{\prime }}_{k^{\prime },m}{D^{\prime }}_{k^{\prime },m}^{*}+{H^{\prime }}_{k^{\prime },m}{B^{\prime }}_{k^{\prime },m}^{*}}{2}dk^{\prime }$$ 19

where *D′*_*k′*,*m*_ and *B′*_*k′*,*m*_ correspond to the displacement and magnetic flux eigenfields, respectively, obtained through the constitutive matrix in eq 9. The total EM energy density is then *n*_T_(*x′*) = ∑_*m*_ *n*_*m*_(*x′*).

In order to corroborate the existence of the two topologically distinct phases in our model, we study the transition between positive and negative modulation strength, with α = 0 in eq 1 marking the topological transition point. This is shown in Figure 2a,b, where we represent the distribution of the energy density of the first band *m* = 1 along the unit cell for both phases. Indeed, we find that for α > 0 the energy density of eq 19 is localized at the center of the unit cell, corresponding to the trivial phase, whereas for α < 0 it shifts to the edges, which marks the obstructed (topological) phase [see Figure 2b]. We note that a subtlety arises in the calculation of θ_*m*_^ST^ for the first band *m* = 1 near the singular point *k′* = ω*′* = 0, where the parity of the electric and magnetic fields becomes ill-defined. Even though this does not affect the quantization of the Zak phase, it obscures the direct correspondence with the Wannier centers (see SI). Crucially, only the relative difference of Zak phases between crystals matters for the appearance of interface states,^75,76^ so this subtlety does not affect the topological characterization of STPhCs.

**Figure 2:**
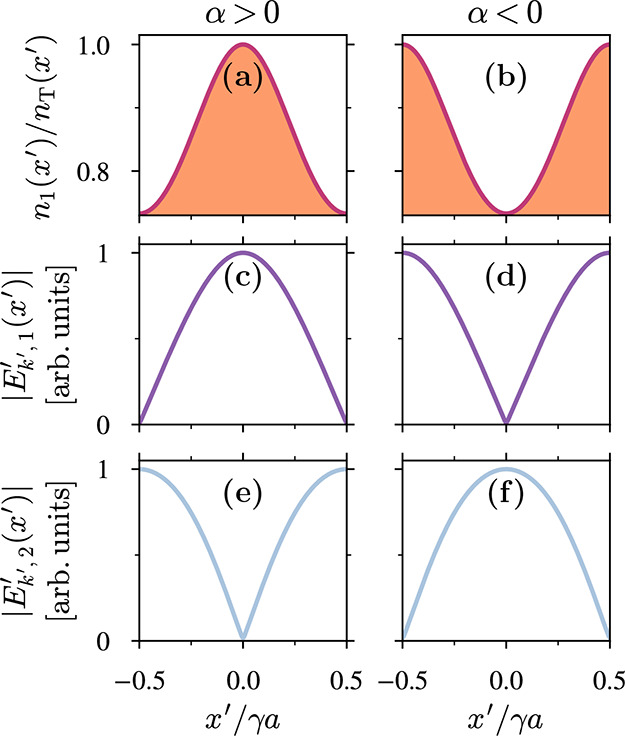
Distinct topological phases in STPhCs. (a, b) Energy density distribution over the unit cell of the first band *m* = 1, for α > 0 and α < 0. (c, d) Absolute value of the electric field eigenfunction $\left|{E^{\prime }}_{k_{\mathrm{gap}}^{\prime },m=1}(x^{\prime })\right|$ located at the first band gap shown in Figure 1d for *k′* < 0, considering α > 0 and α < 0. (e, f) $\left|{E^{\prime }}_{k_{\mathrm{gap}}^{\prime },m=2}(x^{\prime })\right|$ at the same gap for the second band *m* = 2, considering α > 0 and α < 0. All the parameters are the same as in Figure 1.

In Figure 2c–f, we further represent the electric eigenfields at the gap between the first and second bands for both signs of α. The eigenfunctions clearly interchange across the α = 0 transition, a hallmark of band inversion that, as established in ref 77, characterizes a topological phase transition even in non-Hermitian systems as long as they remain in the $P^{\prime }T^{\prime }$ -exact phase. Taken together, these results confirm that $P^{\prime }T^{\prime }$ -symmetry alone suffices to topologically classify subluminal STPhCs, ensuring the existence of two distinct phases even in the absence of parity symmetry.

### Interface State

Building on the prediction of two topologically distinct phases, we now employ a semianalytical scattering matrix formalism (detailed in the SI) to investigate the presence of interface states at the boundary between two subluminal STPhCs, as well as discuss their novel properties due to the temporal modulation. To do so, we consider two types of slabs by truncating the same STPhC in different space-time directions: along the modulation (subluminal ST boundary) [see Figure 3a] and at a fixed position in space (spatial boundary) [see Figure 3f].

**Figure 3:**
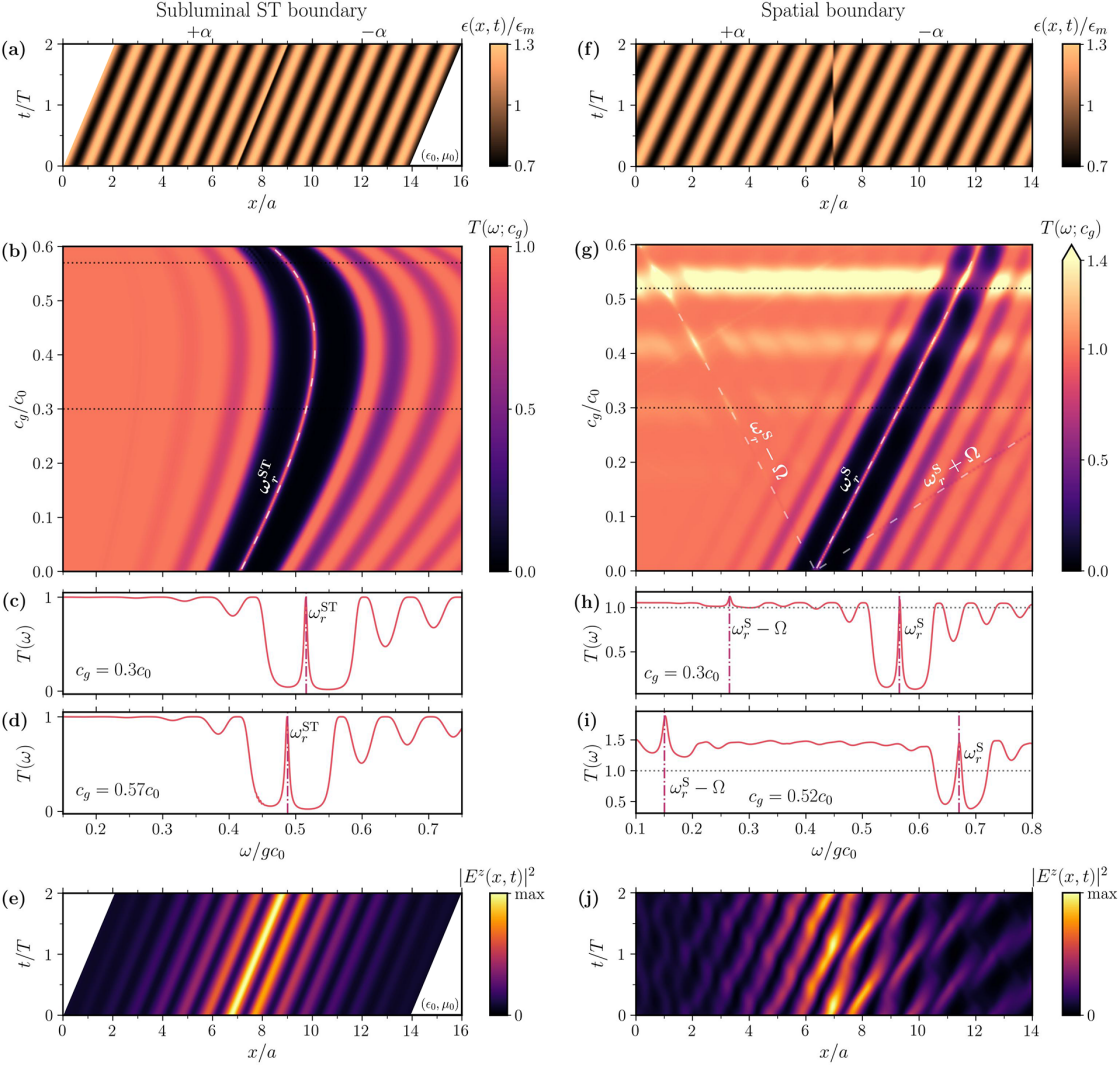
Interface states between a trivial (α > 0) and a topological (α < 0) spatiotemporal slab, considering (a)–(e) a spatiotemporal boundary and (f–j) a spatial boundary. (a) Permittivity profile ϵ­(*x*, *t*) of the two-slab configuration for *c*_g_ = 0.3*c*_0_. (b) Transmittance spectrum of two spatiotemporal slabs whose boundary moves along with the modulation, as a function of the modulation speed *c*_g_. An interface state with resonance frequency following the bulk-predicted ω_r_^ST^ is observed. (c) Horizontal cross section of the transmittance at *c*_g_ = 0.3*c*_0_. A clear peak inside the gap corresponding to the interface state is visible. (d) Same magnitude for *c*_g_ = 0.57*c*_0_. (e) Intensity distribution of the interface state observed when exciting the left slab with a plane wave of frequency ω_r_^ST^. A propagating interface state is observed. (f) Permittivity profile ϵ­(*x*, *t*) of the two spatial slabs for *c*_g_ = 0.3*c*_0_. (g) Transmittance spectrum as a function of *c*_g_ for two slabs of the STPhC with purely spatial boundaries. An interface state is also present for the whole modulation interval. Furthermore, the frequency conversion induced by the temporal modulation enables replicas of the interface states at ω_r_^S^ ± Ω. (h) Horizontal cross section of the transmittance for *c*_g_ = 0.3*c*_0_, revealing the interface state inside the gap as well as the replicas at ω_r_^S^ – Ω. (i) Same magnitude for *c*_g_ = 0.52*c*_0_, where broadband amplification (*T*(ω) > 1) is observed. (j) Intensity distribution of the interface state for a static boundary, showing no propagation.

#### Subluminal Spatiotemporal Interface

We begin by considering two ST slabs, each composed of 7 unit cells of the STPhC defined in eq 1, as represented in Figure 3a. Specifically, we set the medium’s parameters to ϵ_*m*_ = μ_*m*_ = 1.2 and α = 0.3 as in Figures 1 and 2, with different signs of the modulation strength α for each slab. As discussed in the previous section, positive and negative values of α correspond to different topological phases while presenting the same band structure. Consequently, interface states are expected to appear. In Figure 3b, we show the transmission spectrum of this configuration as a function of the grating speed *c*_g_, obtained by exciting the left slab with a plane wave and measuring the transmitted wave at the right end. Horizontal cross sections at *c*_g_ = 0.3*c*_0_ and *c*_g_ = 0.57*c*_0_ are presented in Figure 3c,d, respectively. We observe that the darker regions in Figure 3a, corresponding to lower transmission, mark the band gap, while the continuous peak within the gap identifies the interface state. Its persistence across the entire modulation range confirms the prediction of the ST Zak phase and supports the conclusion that $P^{\prime }T^{\prime }$ -symmetry alone suffices for the topological characterization of the system. Indeed, despite the progressive breaking of $P^{\prime }$ -symmetry with increasing magneto-electric coupling *ξ′*(*x′*;*c*_g_), the interface state persists. We note that all the phenomenology discussed in this section is also present when the incident wave excites the composite system in the opposite direction. More interestingly, the band gap, and therefore the interface state, are observed at lower frequencies due to the nonreciprocal nature of the band structure of STPhCs.

Furthermore, we can predict the resonance frequency ω_r_^ST^ at which interface states will be excited for each *c*_g_ through the following intuition: a topological transition necessarily entails a band gap closing, so the frequency at which this occurs identifies the precise point in the band structure where an interface state must emerge between materials at opposite sides of this transition. Therefore, the resonance frequency can be determined by finding the band-crossing position in the unmodulated case α = 0, which marks the transition point.

In order to find the band crossing point that we argue predicts the resonance frequency of the interface state, we use homogenization theory to obtain a linear approximation of the band dispersion of the STPhC. The dispersion is constructed by copying replicas of the ω = ± *v*_eff_ *k* curves, where $v_{\mathrm{eff}}=1/\sqrt{\epsilon_{\mathrm{eff}}\mu_{\mathrm{eff}}}$ corresponds to the effective velocity defined by the homogenized material parameters (see ref 59). These replicas are translated by the reciprocal lattice vector **p** = (*g*, Ω) an integer number of times *n*, yielding ω + *n*Ω = ± *v*_eff_(*k* + *ng*). Then, calculating the crossing point between the fundamental (*n* = 0) forward mode, and the first replica of the backward mode (*n* = −1), we obtain the crossing point corresponding to the first gap in the forward direction (see sketch in SI):

$$\frac{\omega_{\mathrm{gap}}}{gc_{0}}=\frac{c_{g}+v_{\mathrm{eff}}(c_{g})}{2c_{0}}$$ 20

However, since we are considering a ST interface, it is essential to account for the modified conservation law: it is ω*′* (the comoving frequency) that is conserved, rather than ω. This means that, when a monochromatic wave of frequency ω_0_ encounters the first interface coming from free space, it will not couple to the same frequency inside the material ω_mat_, but rather to a Doppler-shifted frequency given by the expression^9^

$$\omega^{\prime }/\gamma =(1-c_{g}/v_{\mathrm{eff}})\omega_{\mathrm{mat}}=(1-c_{g}/c_{0})\omega_{0}$$ 21

This explains why the band gap position in Figure 3b does not grow linearly with *c*_g_, as might be expected from the tilt induced by the reciprocal lattice vector, but instead shifts to lower frequencies once the modulation speed becomes sufficiently large. This red shift effect can be clearly seen by comparing the transmission spectra for *c*_g_ = 0.3*c*_0_ [Figure 3c] and *c*_g_ = 0.57*c*_0_ [Figure 3d]. As expressed in eq 21, when *c*_0_ ≠ *v*_eff_ the mismatch between ω_0_ and ω_mat_ grows with increasing *c*_g_, leading to the more intricate dependence observed in Figure 3b. Interestingly, this effect makes the gap felt by the incoming wave larger than the width of the actual band gap. Furthermore, from eq 21 we can directly determine the incident frequency necessary to excite ω_gap_ inside the material, which corresponds to the resonance frequency of the topological states at a ST interface:

$$\omega_{r}^{\mathrm{ST}}=\frac{1-c_{g}/v_{\mathrm{eff}}}{1-c_{g}/c_{0}}\omega_{\mathrm{gap}}$$ 22

This frequency is shown as a function of *c*_g_ in Figure 3b with a dashed line, and, as we can see, it predicts with high accuracy the numerically calculated position of the interface state within the gap, showing the connection of these states to the properties of the bulk. This connection, together with the persistence of the interface states, establishes a ST analogue of the bulk-interface correspondence of 1D spatial PhCs.

Finally, we present in Figure 3e the intensity distribution of the interface state excited by a plane wave at its resonance frequency ω_r_^ST^, considering a grating velocity *c*_g_ = 0.3*c*_0_. As observed, the topological state remains localized, decaying evanescently away from the moving boundary. Interestingly, this creates a 1D propagating interface state, in contrast to the 0D static state typically found in 1D spatial PhCs. We note that such a propagation of the interface state at a subluminal spatiotemporal interface can be understood from the tilted nature of the band structure discussed in Figure 1b. Indeed, for a spatial PhC and stationary boundaries, the interface state appears in the band structure of the composite system as a flat band connecting the two edges of the BZ.^58^ Therefore, in a STPhC with ST boundaries, the topological state would also appear joining the two edges of the BZ, which, given the tilted character of the band structure, would grant the state a nonzero group velocity that exactly matches the boundary speed *c*_g_.

#### Spatial Interface

We now turn to the case of two spatial slabs of the same STPhC, as illustrated in Figure 3f, in order to examine how changing the type of boundary affects the interface state. The static interface implies that the slabs are constructed with a purely spatial unit cell, defined with the same spatial period as the modulation, *a* = 2π/*g*. Since this does not correspond to the ST unit cell used to characterize the infinite crystal, the derivation of the scattering matrix for this case needs a tailored approach (see SI). As for the ST interface, we plot in Figure 3g the transmittance spectrum of the slabs in this configuration as a function of the modulation velocity, together with two horizontal cross sections at *c*_g_ = 0.3*c*_0_ and *c*_g_ = 0.52*c*_0_. Crucially, we find that an interface state also appears inside the gap regardless of the value of the grating speed. This demonstrates the robustness and predictive power of the ST Zak phase, which correctly anticipates the existence of such states for two very different types of boundaries. Furthermore, since the spatial interface conserves the lab-frame frequency ω, we can directly predict the resonance frequency of this state through eq 20 as ω_r_^S^ = ω_gap_, without the need for any Doppler shift correction as for the ST boundary case.

From Figure 3g, we observe two key differences from the ST boundary case: (i) the appearance of replicas of the interface state at frequencies that lie outside the band gap, highlighted by dashed lines, and (ii) the amplification of the transmitted wave with *T*(ω) > 1.

First, as reported in ref 58. for a ST phononic crystal, the temporal modulation enables the excitation of the interface state from frequencies shifted in multiples of Ω thanks to the frequency conversion processes occurring at each boundary. This is confirmed by the appearance of a second-order transmission peak at ω_r_^S^ – Ω in Figure 3h,i, highlighted by a vertical line. To understand why this frequency conversion only happens for spatial slabs, we again put our attention on the conservation law for each boundary: conservation of the incident lab-frame frequency ω implies that an incoming plane wave excites all the modes of the band structure that lay in the horizontal line defined by a given ω_0_, whereas conservation of ω*′* implies exciting all the modes laying in a diagonal line with slope equal to *c*_g_.^9^ As a consequence, once we fold each mode into the BZ by shifting them an integer number of reciprocal vectors **p**, we find that the spatial boundary excites an infinite number of modes inside the BZ with frequencies that differ an integer number of Ω with respect to ω_0_.^12^ However, for the ST boundary, each mode along the diagonal line folds back into the same mode inside the BZ, such that we can only excite one truly distinct mode inside the material, explaining why we do not observe frequency conversion in this configuration. We have verified this by means of our semianalytical scattering matrix formalism, which allows us to study the mode decomposition of the scattered fields, as well as the fields inside the slabs (see SI).

Second, the amplification of the transmitted wave can be clearly observed by the horizontal bands of transmittance larger than one in Figure 3g, together with Figure 3i, where most of the transmission spectrum presents *T*(ω) > 1, highlighting the broadband nature of this effect. Within our framework, we can identify frequency conversion processes as the underlying mechanism behind the amplification. Indeed, as shown in the SI, the permittivity encountered by the wave at the second and third interfaces determines whether higher-frequency modes are created in the scattering processes. For a given size of the slab, this condition is satisfied depending on the modulation velocity, explaining the periodic character of the amplification. Moreover, the amplification increases with *c*_g_, since the wave experiences more modulation cycles while traversing the finite slab, allowing for a larger energy transfer from the temporally modulated medium to the wave. In contrast, this effect is absent at ST boundaries, where frequency conversion does not occur. Crucially, this mechanism enables amplification even in the subluminal regime, without the need for the opening of a momentum gap as in PTCs,^78^ facilitating potential experimental realizations with a reduced modulation speed.

Finally, we plot in Figure 3j the interface state excited by an incoming plane wave of frequency ω_r_^S^, considering a modulation velocity *c*_g_ = 0.3*c*_0_. As we can see, although the overall intensity profile presents a similar spatial structure to Figure 3e, the peak of the field’s intensity remains pinned at the static interface, and the state does not propagate without perturbation along the boundary, in contrast to the ST boundary case.

### Robustness against Perturbations

An important aspect of interface states of topological origin is their inherent robustness against certain perturbations. In the previous section, we showed that the interface state presents robustness against an increase in the symmetry-breaking parameter ξ*′*. Indeed, not only did the states persist, but their resonance frequency remained pinned to the one predicted by the band-crossing position ω_r_^ST/S^. Here, we extend this analysis by modifying the properties of one of the slabs, such that the two are no longer related by a simple spatial shift. Moreover, we also discuss the effect of truncating the crystal at arbitrary points rather than at the inversion centers. These configurations represent more general and realistic scenarios, allowing us to test whether the robustness of the interface states extends beyond idealized conditions that might not be met in experimental realizations.

Specifically, we revisit the configuration of ST slabs of Figure 3a, introducing a perturbation Δ that increases the modulation strength of the second slab to α + Δ. In Figure 4a, we evaluate numerically the resonance frequency ω_*i*_ of the interface state and plot it as a function of Δ and *c*_g_ to study its deviation from the bulk prediction ω_r_^ST^. Importantly, while this frequency plays a role similar to the midgap position in chiral-symmetric systems, here a deviation does not always signal a loss of bulk connection, and thus the source of the deviation needs to be carefully studied. In the standard case of a spatial 1D PhC, *c*_g_ = 0, we observe no deviation with Δ, highlighting the robustness of the interface states, since they are completely insensitive to the perturbation. As we turn on and increase the modulation speed, however, the asymmetry in the properties of each slab becomes more relevant, as the deviation grows with increasing Δ and *c*_g_. This effect is clearly observed when comparing the position of the transmission peaks for *c*_g_ = 0.1*c*_0_ and *c*_g_ = 0.5*c*_0_ in Figure 4b,c, respectively.

**Figure 4:**
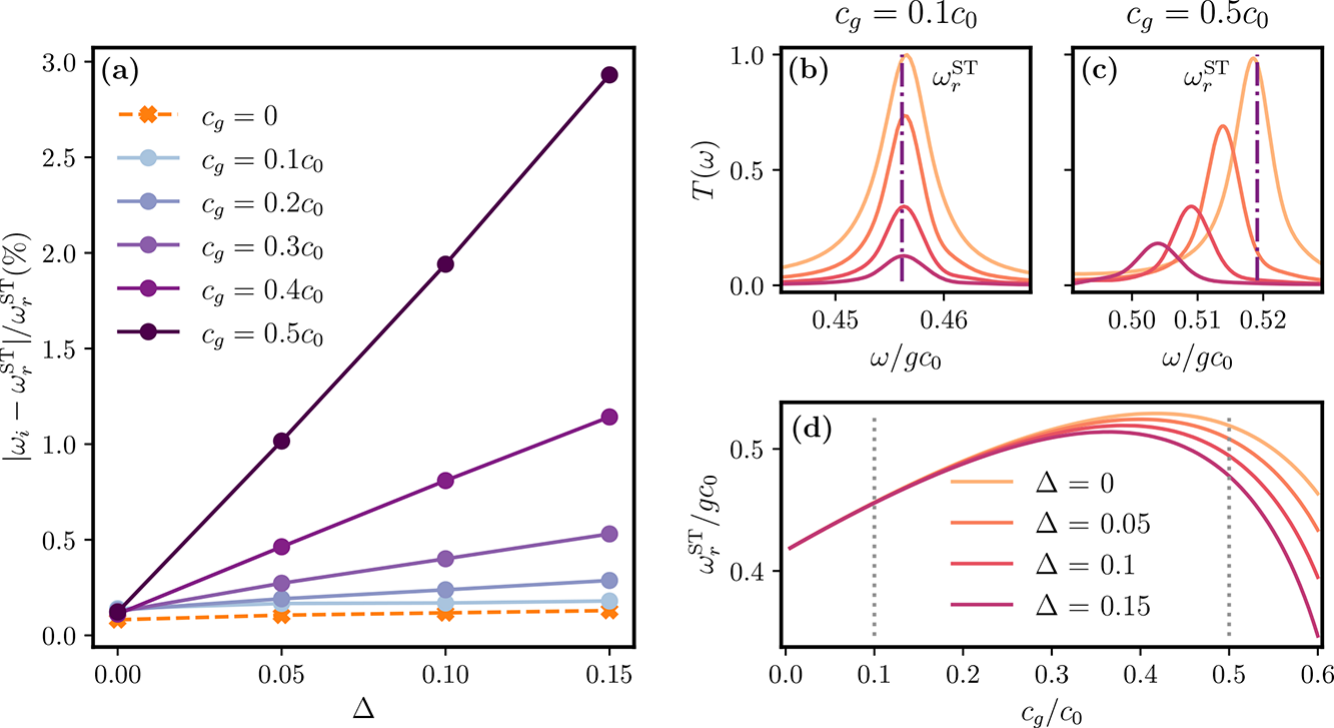
Robustness of interface states studied in the two-slab configuration of Figure 3a by perturbing the system with an increased modulation strength in the second slab by (α + Δ), with Δ the perturbation parameter. (a) Deviation of the numerically obtained resonance frequency ω_*i*_ from the bulk-predicted ω_r_^ST^ as we increase Δ and *c*_g_. (b) Zoom in at the peaks of transmittance observed inside the gap for *c*_g_ = 0.1*c*_0_, with a vertical line showing ω_r_^ST^ as a reference. (c) Same magnitude for *c*_g_ = 0.5*c*_0_. (d) Analytical resonance frequency ω_r_^ST^ for different modulation strengths (α + Δ) as a function of the modulation speed *c*_g_.

The deviation can be understood by noting that the effective parameters (ϵ_eff_, μ_eff_) used to derive the band-crossing position depend on the modulation strength when *c*_g_ ≠ 0.^59^ Indeed, Figure 4d shows how the predicted resonance frequency ω_r_^ST^ for a STPhC with modulation strength α + Δ splits into distinct values for different Δ as the modulation speed increases. Consequently, in a finite system composed of two slabs with different modulation strengths, each slab predicts a distinct ω_r_^ST^, explaining the deviation and the apparent loss of predictive power. Crucially, however, this does not imply a breakdown of bulk-interface correspondence, but rather highlights the need for a more refined bulk theory to determine the exact band-crossing position in such asymmetric slab configurations. To emphasize this point, we note that the deviation in frequency observed in Figure 4 is therefore different in nature from the one that would induce a phase perturbation ϕ in the modulation eq 1. Indeed, such a shift truncates the slabs at points different from the inversion centers of the unit cells, which obscures the distinction between trivial and topological phases by lifting the quantization of the Zak phase. While interface states may still appear in that configuration, as reported for the static case in refs 79 and 80 , their resonance frequency cannot be related anymore to the bulk-predicted value, signaling in that case a loss of topological nature.

### Superluminal Regime

Once we have characterized the topology of STPhCs in the subluminal regime, we now extend our formalism to investigate the interface states of superluminal STPhCs. To do so, we briefly discuss the main differences between regimes, and then establish the new reference frame in which we study the relevant symmetries and define the corresponding topological invariant.

Figure 5a shows the permittivity distribution for a modulation speed of *c*_g_ = 5*c*_0_. As in the subluminal case, the system remains effectively 1D, exhibiting a moving grating profile and a continuous symmetry along the space-time direction. A key difference, however, is that although the reciprocal lattice vector is still given by **p** = (*g*, Ω), the lattice vector **L** now has a larger temporal than spatial component, hinting at the time-like nature of the system. This feature is confirmed when we study its band structure, shown in Figure 5b, where momentum gaps (*k*-gaps) with complex frequency appear, analogous to those found in PTCs. Recently, these *k*-gaps have attracted special attention due to their ability to amplify an incoming wave in time, as the usual energy conservation constraint is lifted.^1^ Furthermore, contrary to PTCs, the *k*-gaps found in superluminal STPhCs are asymmetric, which further enriches this phenomenology by enabling unidirectional amplification.

**Figure 5:**
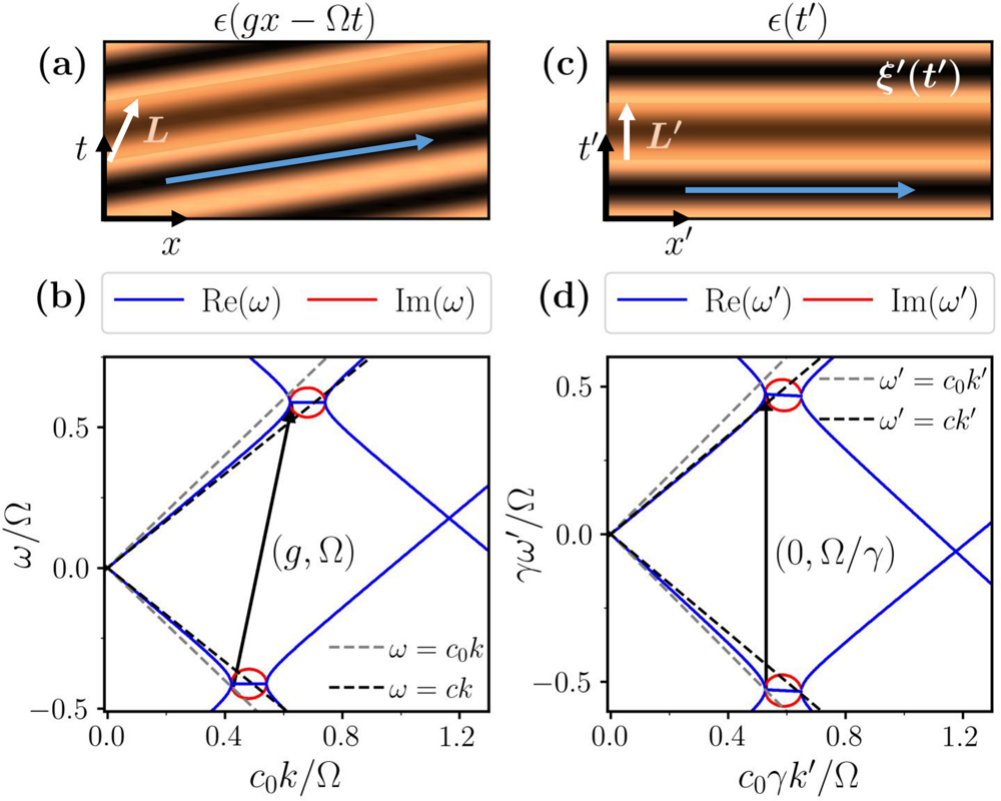
Spatiotemporal photonic crystal in the laboratory and time-like frames for superluminal modulation. (a) Traveling-wave modulated permittivity in the lab-frame. A blue arrow signals the direction of the continuous space-time translation symmetry that defines the unit cell highlighted in the orange shaded region. The white arrow corresponds to the lattice vector. (b) Band structure of a STPhC for a modulation speed *c*_g_ = 5*c*_0_, modulation strength α = 0.4 and ϵ_*m*_ = μ_*m*_ = 1.2. Gray and black dashed lines correspond to the dispersion relation in free space and in the unmodulated material, respectively. Black arrow represents the reciprocal lattice vector **p**. (c) Modulated permittivity in the time-like frame. The previous magnitudes are now temporal-like and a magneto-electric coupling ξ*′*(*x′*) appears. (d) Band structure of the same STPhC in the comoving frame, with the black arrow now representing the transformed reciprocal vector **p** *′*.

In order to apply the same methodology as in the subluminal case, we need to make a change of the reference frame such that its magnitudes and symmetries are purely temporal or spatial. In this case, however, we cannot make the same transformation to a frame that comoves with the modulation, since the Lorentz factor γ would give unphysical imaginary values due to the modulation speed being higher than the speed of light. Instead, given the temporal character of the band structure, we want to turn the modulation in the transformed frame purely temporal. A transformation into such a time-like frame is achieved by setting the frame velocity in the Lorentz transformation from eqs 6 and 7 equal to^9^

$$c_{f}=c_{0}^{2}/c_{g}$$ 23

Crucially, as it remains a Lorentz transformation, the structure of Maxwell’s equations, as well as the expressions for the constitutive parameters, are the same as in the subluminal regime [eqs 8–12] with the modulation speed *c*_g_ changed to *c*_f_.

Figure 5c shows the permittivity distribution as seen in the time-like frame, only periodic in *t′* and presenting a moving-medium type magneto-electric coupling ξ*′*(*t′*). In this frame, the unit cell is defined in the temporal variable as *t′* ∈ [−γ*T*/2, γ*T*/2], with a lattice vector **L** *′* = [0, γ*T*], where *T* = 2π/Ω is the temporal period. Furthermore, we can use eqs 13 and 14 with *c*_f_ to transform the reciprocal variables in the new frame (*k′*, ω*′*) and obtain the transformed reciprocal vector **p** *′* = [0, Ω/γ], as well as the time-like-frame BZ ω*′* ∈ [−Ω/2γ, Ω/2γ]. Finally, making the inverse transformation, we obtain the lattice vector in the lab-frame as **L** = γ^2^ *T* [*c*_f_, 1] = γ^2^ *T* [*c*_0_^2^/*c*_g_, 1].

As we can see, an important consequence of the periodicity being present in time is that the BZ is now defined in the frequency dimension, similarly to Floquet systems.^50,51^ Therefore, to obtain the eigenfunctions in the time-like frame, we derive an eigenvalue equation for *k′*. Due to the temporal dependence of the constitutive matrix, eq 8 takes the form

$$\left(\begin{array}{ll} 0 & \partial_{x^{\prime }}\\ \partial_{x^{\prime }} & 0 \end{array}\right)\left[\begin{array}{l} {E_{z}}^{\prime }\\ {H_{y}}^{\prime } \end{array}\right]=\partial_{t^{\prime }}\left({\mathrm{M̂}}^{\prime }(t^{\prime })\left[\begin{array}{l} {E_{z}}^{\prime }\\ {H_{y}}^{\prime } \end{array}\right]\right)$$ 24

Using eq 4, we arrive at the following expression

$${\mathrm{M̂}}^{\prime }(t^{\prime })\left(\begin{array}{ll} 0 & \partial_{t^{\prime }}\\ \partial_{t^{\prime }} & 0 \end{array}\right)\left[\begin{array}{l} {D_{z}}^{\prime }\\ {B_{y}}^{\prime } \end{array}\right]=\partial_{x^{\prime }}\left[\begin{array}{l} {D_{z}}^{\prime }\\ {B_{y}}^{\prime } \end{array}\right]$$ 25

where we clearly see that [*D*_*z*_^′^, *B*_*y*_^′^]^*T*^ corresponds to the natural choice of eigenfunctions in the time-like frame. Finally, making use of the temporal periodicity, we apply a Floquet’s ansatz to eq 25 to obtain the dispersion relation *k′*(ω*′*), together with the time-like frame eigenfunctions

$${\left[\begin{array}{l} D^{\prime }(t^{\prime })\\ B^{\prime }(t^{\prime }) \end{array}\right]}_{\omega^{\prime },m}=e^{-i\omega^{\prime }t^{\prime }}{\left[\begin{array}{l} {u^{\prime }}^{D}(t^{\prime })\\ {u^{\prime }}^{B}(t^{\prime }) \end{array}\right]}_{\omega^{\prime },m}$$ 26

where *u*_ω′,*m*_^*D*/*B*^(*t′* + γ*T*) = *u*_ω′,*m*_^*D*/*B*^(*t′*) correspond to the periodic part of the eigenfields, and *m* represents the band index.

The corresponding band dispersion is shown in Figure 5d. As before, the change of reference frame removes the asymmetry in the band gaps, owing to the absence of a momentum component in **p** *′*. However, the system still exhibits nonreciprocal behavior, as *k′*(−ω*′*) ≠ *k′*(ω*′*), highlighting the role of the bianisotropic term ξ*′*(*t′*). Indeed, since Maxwell’s equations in the time-like frame retain the same structure as in the comoving frame of the subluminal case, the system inherits the same symmetry properties in the new frame. Consequently, even though the constitutive parameters are symmetric under $T^{\prime }$ , the magneto-electric coupling breaks parity and time-reversal symmetry individually, which leads to nonreciprocity.

In this case, however, increasing the modulation speed *c*_g_ does not necessarily lead to stronger symmetry breaking, since the bianisotropic coupling ξ*′*(*t′*) now depends on *c*_f_. In the limit *c*_g_ → *∞*, corresponding to a purely temporal modulation where the system reduces to a PTC, one has *c*_f_ → 0. In this regime, the magneto-electric coupling vanishes, and both parity and time-reversal symmetries are restored. This shows that, in either reference frame, it is the proximity to the luminal regime that induces the symmetry breaking rather than an increase in the modulation speed.

#### Topological Characterization

Importantly, $P^{\prime }T^{\prime }$ -symmetry is always preserved. Building on the topological framework developed for subluminal STPhCs, we examine whether a symmetry-protected topological invariant can also be defined through this symmetry in a system with predominantly temporal character. To this end, we evaluate the Zak phase using the eigenfunctions of eq 26 and integrate it over the BZ defined in the time-like frame

$$\theta_{m}^{\mathrm{ST}}=i\int_{-\Omega /2\gamma }^{+\Omega /2\gamma }d\omega^{\prime }\langle {u^{\prime }}_{\omega^{\prime },m}|\partial_{\omega^{\prime }}{u^{\prime }}_{\omega^{\prime },m}\rangle_{({\mathrm{M̂}}^{\prime }{)}^{-1}}$$ 27

where the integrand corresponds to the Berry connection and **u**_ω*′*,*m*_ = [*u′*_ω*′*,*m*_^*D*^, *u′*_ω*′*,*m*_^*B*^]^*T*^. Crucially, even if the system is clearly non-Hermitian due to its temporal modulation, we can also relate the left and right eigenvectors of the system in the time-like frame thanks to the Hermiticity of the constitutive matrix and $P^{\prime }T^{\prime }$ symmetry (see SI). As a result, we can use the conventional Berry connection defined with a weighted scalar product over the unit cell given by

$$\left\langle {\Psi^{\prime }}_{1}|{\Psi^{\prime }}_{2}\rangle_{({\mathrm{M̂}}^{\prime }{)}^{-1}}=\int_{-\gamma T/2}^{+\gamma T/2}dt^{\prime }{{\Psi_{1}}^{\prime }}^{†}(t^{\prime })(\mathrm{M̂}(t^{\prime }){)}^{-1}{\Psi^{\prime }}_{2}(t^{\prime }\right)$$ 28

with **Ψ** *′*_ω*′*,*m*_ = [*D′*_ω*′*,*m*_, *B′*_ω*′*,*m*_]^*T*^ the time-like-frame eigenfunctions, normalized by establishing ⟨**Ψ** *′*_ω*′*,*m*_|**Ψ** *′*_ω*′*,*m*_⟩_(**M̂** *′*)^−1^_ = 1. Therefore, since we are able to write the ST Zak phase with a Hermitian Berry connection, we can make use of the results of ref 70. to conclude that $P^{\prime }T^{\prime }$ -symmetry alone allows the quantization of the ST Zak phase defined in eq 27. With this, we have derived a $Z_{2}$ topological invariant for superluminal STPhCs, extending our topological characterization of STPhCs to its whole modulation regime. This result unifies a wide class of systems, ranging from spatial PhCs to PTCs, and brings further light into why the Hermitian definition of the Zak phase was able to characterize the topology of PTCs’ band structures in previous works.^45^

Building on this result, we now investigate the existence of interface states between two superluminal STPhC slabs characterized by different ST Zak phases. To do so, we consider two types of truncations: a spatiotemporal boundary that follows the space-time symmetry highlighted in Figure 5a, and a purely temporal boundary.

These configurations correspond to the temporal counterparts of the cases studied in the subluminal regime. However, the temporal nature of the boundaries introduces important differences. First, frequency is no longer conserved, but rather momentum is: *k′* for spatiotemporal boundaries and *k* for purely temporal ones. Second, temporal reflection is fundamentally different from a conventional spatial reflection. Due to causality, a temporally reflected wave cannot propagate within the same space-time region as the incident wave, as this would imply backward propagation in time. Instead, both reflected and transmitted waves remain in the same medium after the boundary, but each one propagates in a different spatial direction. Taking these considerations into account, we generalize our scattering-matrix formalism to compute the interface states in superluminal STPhCs and explore their distinctive features (see SI).

#### Superluminal Spatiotemporal Interface

We first consider two slabs, each composed of 15 ST unit cells, with the same parameters as in Figure 5. To visualize the composite system in a space-time diagram analogous to the subluminal case [Figure 3a], one may invoke the space-time duality between modulation regimes. In particular, subluminal and superluminal systems can be related by reflecting the slabs along the light line.^9^ Under this transformation, the permittivity distribution of the two superluminal slabs can be visualized by interchanging the spatial and temporal axes in Figure 3a. A subtlety arises, however, when considering the wave excitation of the composite system. Since the velocity of the slab exceeds the wave velocity, the usual picture of a wave impinging on a boundary no longer applies. Instead, in order to excite the finite system, it is the slab that sweeps over the wave.

Figure 6a shows the transmittance spectrum of the two ST slabs as a function of the inverse modulation speed, *c*_0_/*c*_g_ = *c*_f_/*c*_0_, obtained by exciting the composite system with plane waves of momentum *k* at normal incidence. An horizontal cross section at the PTC limit is shown in Figure 6b, presenting remarkable amplification of the transmitted wave due to the complex frequency modes in the *k*-gaps as well as a dip in transmission. Interestingly, we note that this dip has been predicted and observed experimentally in PTCs, as well as its associated interface states localized in time.^81,82^ In our case, we observe such a dip in transmission throughout the whole modulation regime, therefore confirming the existence of robust interface states in superluminal STPhCs.

**Figure 6:**
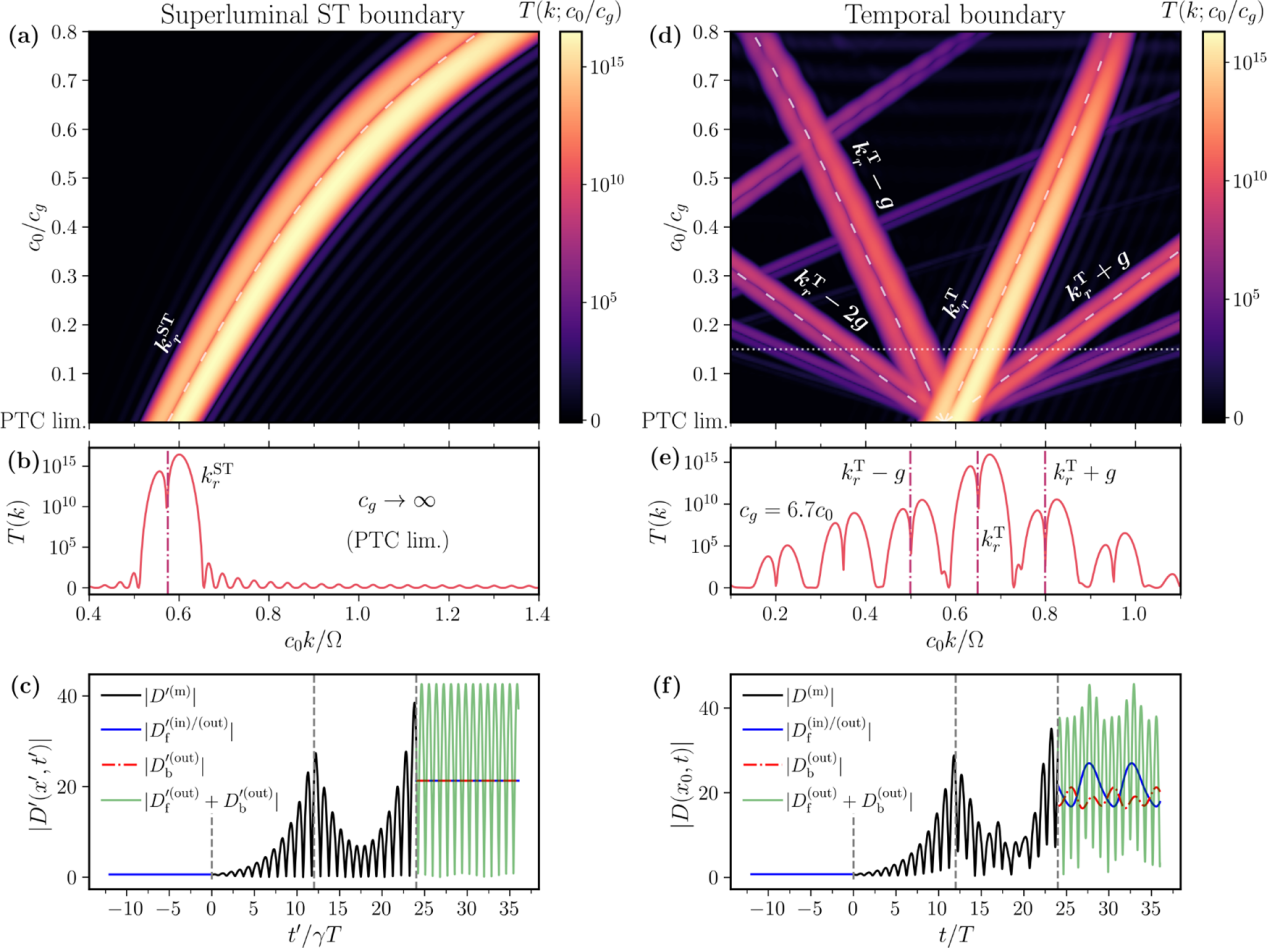
Interface states in the superluminal regime between a trivial (α > 0) and a topological (α < 0) spatiotemporal slab, considering (a–c) a spatiotemporal boundary and (d–f) a temporal boundary. (a) Transmittance spectrum of two spatiotemporal slabs whose boundary moves along with the modulation, as a function of *c*_0_/*c*_g_. An interface state with resonance frequency following the bulk-predicted *k*_r_^ST^ is observed. (b) Horizontal cross section of the transmittance at the PTC limit *c*_g_ → *∞*. A clear dip inside the amplifying gap corresponding to the interface state is visible. (c) Absolute value of the displacement field for *c*_g_ = 5*c*_0_ and 12 unit cells per slab as a function of time at every region of space-time delimited by gray dashed lines: vacuum before the slabs (*D*_f_′^(in)^), inside the two STPhC slabs (*D′* ^(m)^), and vacuum after the slabs (*D*_f/b_^′(out)^). Localization at the temporal interface is observed. (d) Transmittance spectrum as a function of *c*_0_/*c*_g_ for two slabs of STPhC with purely temporal boundaries. An interface state, as well as its replicas at *k*_r_^S^ ± *mg* due to momentum conversion, are observed similar to the subluminal case. (e) Horizontal cross section of the transmittance for *c*_g_ = 6.7*c*_0_, revealing the interface state inside the gap as well as the many replicas. (f) Absolute value of the fields at different regions of space-time. Temporal dependence of the transmitted wave shows the presence of different harmonic modes, excited due to momentum conversion.

Furthermore, we can relate the position of the boundary state through the band-crossing point derived for the subluminal regime in eq 20 with

$$k_{\mathrm{gap}}=\omega_{\mathrm{gap}}/v_{\mathrm{eff}}$$ 29

Then, since we need to account for the conservation of *k′*, we know that the incident wave with *k*_0_ will couple to a mode inside the material with momentum *k*_mat_ given by

$$k^{\prime }/\gamma =(1-c_{f}/v_{\mathrm{eff}})k_{\mathrm{mat}}=(1-c_{f}/c_{0})k_{0}$$ 30

Again, the enforcement of this modified conservation law explains why the position of the band gap does not grow linearly with *c*_f_, but rather presents a more intricate dependence. Taking this into account, we can derive an analytical expression for the resonant momentum of the interface state as

$$k_{r}^{\mathrm{ST}}=\frac{1-c_{f}/v_{\mathrm{eff}}}{1-c_{f}/c_{0}}k_{\mathrm{gap}}$$ 31

This magnitude is represented as a function of *c*_0_/*c*_g_ in Figure 6a with a dashed line. As we can see, it perfectly follows the dip in transmission, which confirms the topological nature of the state in the superluminal regime.

Finally, in Figure 6c we plot the field distribution of the interface state |*D*_*z*_ *′*(*x′*, *t′*)| in the time-like frame across each region of the composite system, delimited by gray dashed lines: vacuum before the slabs (*D*_f_ *′* ^(in)^), inside the positive and negative modulation strength α slabs (*D*′^(m)^), and vacuum after the slabs (*D*_f/b_^′(out)^). As shown, the state is pinned to the temporal interface *t′* = 12γ*T*, where it presents a sharp decay within an otherwise amplifying medium. As a result, the field amplitude grows again until it reaches the third boundary, which leads to reduced amplification of the transmitted wave. This behavior explains why the interface state manifests as a dip in the transmittance spectrum. In the time-like frame, the localization in the transformed time *t′* is analogous to the interface states observed in PTCs. However, upon transformation back to the lab frame, we see the state propagating at a superluminal speed together with the moving boundary. Finally, beyond the slabs, two plane waves with constant absolute values are observed, propagating in opposite directions with an amplified amplitude.

#### Temporal Interface

We now turn to the case of two temporal slabs of the same superluminal STPhC with α of opposite signs, composed of purely temporal unit cells of period *T*, whose permittivity distribution can be obtained by interchanging the spatial and temporal axes of Figure 3f. For this configuration, Figure 6d shows the transmittance spectrum of a forward-propagating incident wave, analogous to the ST-boundary case. A transmission dip is again observed within the *k*-gap throughout the superluminal modulation regime, confirming the presence of interface states. Moreover, since the conserved quantity at a temporal boundary is the standard momentum *k*, the resonant momentum can be accurately predicted from eq 29 as *k*_r_^T^ = *k*_gap_.

More interestingly, the conservation of *k*, together with the tilted band structure shown in Figure 5b, leads to momentum-conversion at each scattering process analogous to the frequency-conversion observed in the subluminal regime. Through the same mechanism, replicas of the *k*-gaps and therefore of the interface states are created. In this regime, these replicas are more pronounced due to the amplifying nature of the gaps, which allows the observation of higher-order excitations. Indeed, Figure 6e presents a horizontal cross section of the transmittance spectrum at *c*_g_ = 6.7*c*_0_, where up to fourth-order replicas can be observed at lower momentum values. Furthermore, since the spacing between replicas depends on the modulation spatial frequency *g*, the gaps can overlap at sufficiently small *g* (high modulation speeds), resulting in broadband amplification that is significantly stronger than that observed in subluminal STPhCs.

Finally, Figure 6f shows the field distribution |*D*_*z*_(*x*_0_, *t*)| of the interface state at a fixed spatial position *x*_0_ in the lab frame. Although the temporal dependence is more intricate, the state remains localized in time at the interface *t* = 12*T*. Beyond the slabs, forward- (*D*_f_^(out)^) and backward-propagating waves (*D*_b_^(out)^) are observed, whose absolute value varies in time. This indicates that both transmitted and reflected fields contain multiple harmonic components, which is a direct consequence of momentum conversion.

## Conclusions

In this work, we investigate the topological origin of interface states in spatiotemporal photonic crystals with traveling-wave modulation and uncover their unique properties. Through the appropriate consideration of the symmetries present in the system, we provide a framework to topologically classify time-dependent modulations of traveling-wave type for both subluminal and superluminal regimes. Notably, we show that computing topological invariants in a frame where the modulation depends on only one transformed variable, either space for the subluminal regime or time for the superluminal regime, leads to a well-defined and meaningful topological classification. Using Lorentz transformations, we show that the spatiotemporal counterpart of parity-time-reversal symmetry is conserved. This symmetry enforces the quantization of the spatiotemporal Zak phase defined along the transformed Brillouin Zone, yielding a $Z_{2}$ topological invariant. By calculating the electromagnetic band energy density, we distinguish the two resulting phases as a trivial phase and an obstructed atomic limit, which clarifies the topological nature of interface states in spatiotemporal photonic crystals without further symmetries. Our formalism holds for arbitrary modulation velocities, bridging a wide range of systems from conventional photonic crystals to photonic time crystals and providing new insights into the topological characterization of the latter.

We prove this point by calculating semianalytically the interface states that arise between slabs of different spatiotemporal Zak phases for different types of boundaries, depending on the modulation regime: spatial, temporal, and spatiotemporal. From the transmittance spectra, we find that, for each boundary, an in-gap state exists in both subluminal and superluminal regimes and that its resonance frequency, or momentum, can be accurately predicted solely from bulk properties. The interface states also present novel features stemming from the nonreciprocal nature of traveling-wave media and the lack of energy conservation. For a spatiotemporal interface, the topological state propagates along with the moving boundary even at superluminal speeds, a remarkable feature in an effectively one-dimensional system. In contrast, both static and temporal interfaces introduce frequency or momentum conversion at each scattering event, producing replicas of the interface states and broadband amplification of the transmitted wave. Furthermore, nonreciprocity in this system also enables selective excitation, which in the superluminal regime gives rise to directional amplification due to the presence of momentum gaps.

Our results highlight the potential of temporal modulation to enrich the properties of interface states in spatiotemporal crystals. This paves the way to explore time-varying effects in higher-dimensional systems with additional symmetries where richer topological phases already exist in the static limit. Although this work focuses on photonic systems, our predictions are general and extend to other wave platforms where spatiotemporal interfaces and traveling-wave modulations have already been experimentally demonstrated: elastic materials such as elastic strips^18^ or piezoelectric crystals,^19^ acoustic systems,^57^ and transmission lines.^25^ This underscores the feasibility of realizing these effects experimentally and broadens their potential applications beyond those of their static counterparts.^83–85^ Altogether, these directions point to a broader landscape in which spatiotemporal modulation becomes a key ingredient for engineering novel topological phases for wave control.

## Supplementary Material



## Notes

The following files are available free of charge. The Supporting Information is available free of charge at https://pubs.acs.org/doi/10.1021/acsphotonics.5c02806.

Detailed derivation of the solutions of Maxwell’s equations for space-time photonic crystals in different reference frames, together with the calculation of the scattering matrix of the finite system for every boundary considered in the text; and derivation of the spatiotemporal Zak phase and a study of the broadband amplification process (PDF)
