# Supplementary material for: Interface States in Space-Time Photonic Crystals: Topological Origin, Propagation, and Amplification
Source: ACS Photonics. 2026 May 21;13(11):3012–25. doi: 10.1021/acsphotonics.5c02806 (PMC13237818; doi:10.1021/acsphotonics.5c02806)
Supplement: Supplementary file 1 [file ph5c02806_si_001.pdf]

Supporting Information - Interface States in Space-Time Photonic Crystals: Topological Origin, Propagation and Amplification

Alejandro Caballero[1,2,*] Thomas F. Allard[1,2], and Paloma A. Huidobro[1,2†]
[1] *Departamento de Física Teórica de la Materia Condensada, Universidad Autónoma de Madrid, E28049 Madrid, Spain and*
[2] *Condensed Matter Physics Center (IFIMAC), Universidad Autónoma de Madrid, E28049 Madrid, Spain*

CONTENTS

I. Maxwell's equations in spatiotemporal photonic crystals S1
A. Eigenvalue problem for the lab-frame S2
B. Eigenvalue problem for the comoving frame S4

II. Scattering matrix and transmission spectrum S6
A. Spatiotemporal Boundary S6
B. Spatial Boundary S9

III. Spatiotemporal Zak phase S9

IV. Band crossing position S10

V. Amplification S10

VI. Superluminal Regime S12
A. Eigenvalue problem for the time-like frame S12
B. Scattering matrix and transmission spectrum S13
1. Spatiotemporal Boundary S13
2. Temporal Boundary S15
C. Spatiotemporal Zak phase S15

References S16

I. MAXWELL'S EQUATIONS IN SPATIOTEMPORAL PHOTONIC CRYSTALS

In this section we detail our approach to solve Maxwell's equations for spatiotemporal photonic crystals (STPhCs). Let us first consider a general scenario where both the permittivity and permeability are modulated in space and time following a travelling-wave:

$$\epsilon(x,t) = \epsilon_0\epsilon_m(1 + 2\alpha_e \cos(gx - \Omega t)), \tag{1}$$

$$\mu(x,t) = \mu_0\mu_m(1 + 2\alpha_m \cos(gx - \Omega t)). \tag{2}$$

Then, we can write Maxwell's equations in the lab-frame as

$$\hat{\mathbf{L}}\mathbf{\Psi} = \partial_t \left(\hat{\mathbf{M}}\mathbf{\Psi}\right), \tag{3}$$

with

$$\hat{\mathbf{L}} = \begin{pmatrix} \mathbb{0}_{3\times3} & \nabla\times \\ -\nabla\times & \mathbb{0}_{3\times3} \end{pmatrix}, \quad \hat{\mathbf{M}}(x,t) = \begin{pmatrix} \epsilon(x,t)\mathbb{1}_{3\times3} & \mathbb{0}_{3\times3} \\ \mathbb{0}_{3\times3} & \mu(x,t)\mathbb{1}_{3\times3} \end{pmatrix}, \quad \mathbf{\Psi} = \begin{bmatrix} \mathbf{E} \\ \mathbf{H} \end{bmatrix}. \tag{4}$$

* alejandro.caballero@uam.es
† p.arroyo-huidobro@uam.es

On the other hand, the spatiotemporal (ST) nature of the system complicates the definition of the relevant symmetries and magnitudes compared to regular photonic crystals. As discussed in the main text, we can overcome this difficulty by performing a Lorentz transformation to the frame comoving with the modulation. Specifically, we apply the Lorentz boost defined in Eqs. (6–7) of the main text, which preserves the form of Maxwell's equations but, crucially, modifies the constitutive relations of the transformed fields. After this transformation, we obtain

$$\hat{\mathbf{L}}'\mathbf{\Psi}' = \hat{\mathbf{M}}'\partial_{t'}\mathbf{\Psi}', \tag{5}$$

with

$$\hat{\mathbf{L}}' = \begin{pmatrix} \mathbb{0}_{3\times3} & \nabla'\times \\ -\nabla'\times & \mathbb{0}_{3\times3} \end{pmatrix}, \quad \hat{\mathbf{M}}'(x') = \begin{pmatrix} \epsilon'_{\parallel}(x') & 0 & 0 & 0 & 0 & 0 \\ 0 & \epsilon'_{\perp}(x') & 0 & 0 & 0 & -\xi'(x') \\ 0 & 0 & \epsilon'_{\perp}(x') & 0 & \xi'(x') & 0 \\ 0 & 0 & 0 & \mu'_{\parallel}(x') & 0 & 0 \\ 0 & 0 & \xi'(x') & 0 & \mu'_{\perp}(x') & 0 \\ 0 & -\xi'(x') & 0 & 0 & 0 & \mu'_{\perp}(x') \end{pmatrix}, \quad \mathbf{\Psi}' = \begin{bmatrix} \mathbf{E}' \\ \mathbf{H}' \end{bmatrix}. \tag{6}$$

The expressions for the components of the constitutive matrix are given in the main text, and $\epsilon'_{\parallel}(x') = \epsilon(x'/\gamma)$, $\mu'_{\parallel}(x') = \mu(x'/\gamma)$. As we can see, a moving medium type bianisotropic coupling between electric and magnetic fields appears.

A. Eigenvalue problem for the lab-frame

As discussed in the main text, Maxwell's equations can be solved by expanding the fields in a Bloch-Floquet form. Considering an s-polarized wave travelling along with the modulation [1, 2],

$$\begin{bmatrix} E_z(x,t) \\ H_y(x,t) \end{bmatrix} = e^{i(kx-\omega t)} \sum_n \begin{bmatrix} E_n \\ H_n \end{bmatrix} e^{i\,n(gx-\Omega t)}, \tag{7}$$

where $-N_{\mathrm{F}} \leq n \leq N_{\mathrm{F}}$, and $2N_{\mathrm{F}}+1$ is the total number of Bloch-Floquet harmonics included in the truncated field expansion. Substituting Eq. (7) into Maxwell's equations yields the following eigenvalue problem for the field Floquet amplitudes:

$$\begin{pmatrix} \mathbb{M}^{\mathrm{EE}}(\omega) & \mathbb{M}^{\mathrm{EH}}(\omega) \\ \mathbb{M}^{\mathrm{HE}}(\omega) & \mathbb{M}^{\mathrm{HH}}(\omega) \end{pmatrix} \begin{bmatrix} \mathbf{E}_\omega \\ \mathbf{H}_\omega \end{bmatrix} = k_\omega \begin{bmatrix} \mathbf{E}_\omega \\ \mathbf{H}_\omega \end{bmatrix}, \tag{8}$$

where each component of the 2×2 block matrix is another square matrix of dimension $(2N_{\mathrm{F}}+1)\times(2N_{\mathrm{F}}+1)$, giving a total of $2(2N_{\mathrm{F}}+1)$ eigenvalues. Since only s-polarization is considered throughout this work, the subscripts y and z are omitted for simplicity. Furthermore, we make the dependence with ω explicit in order to distinguish between solutions in the laboratory and comoving frames, since the latter is defined for a Lorentz transformed frequency ω'.

The row (n) and columns (n') entries of the matrices read:

$$\mathbb{M}^{\mathrm{EE}}_{n,n'} = \mathbb{M}^{\mathrm{HH}}_{n,n'} = -ng\delta_{n,n'}, \tag{9}$$

$$\mathbb{M}^{\mathrm{EH}}_{n,n'} = -\mu_0\mu_m(\omega+n\Omega)[\delta_{n,n'} + \alpha_m(\delta_{n,n'+1} + \delta_{n,n'-1})], \tag{10}$$

$$\mathbb{M}^{\mathrm{HE}}_{n,n'} = -\epsilon_0\epsilon_m(\omega+n\Omega)[\delta_{n,n'} + \alpha_e(\delta_{n,n'+1} + \delta_{n,n'-1})]. \tag{11}$$

Once the eigenproblem is solved, we organize the eigenvectors as follows

$$\mathcal{M}^{(\mathrm{m})}_\omega = \left(\mathbf{\Psi}_{\omega,\tau=1,p=-N_{\mathrm{F}}}, \ldots, \mathbf{\Psi}_{\omega,\tau=1,p=N_{\mathrm{F}}}, \mathbf{\Psi}_{\omega,\tau=2,p=-N_{\mathrm{F}}}, \ldots, \mathbf{\Psi}_{\omega,\tau=2,p=N_{\mathrm{F}}}\right), \tag{12}$$

defining a $2\,(2N_{\mathrm{F}}+1)\times 2\,(2N_{\mathrm{F}}+1)$ matrix whose columns correspond to the eigenvectors, where

$$\mathbf{\Psi}_{\omega,\tau,p} = \left[E_{\omega,\tau,p,n=-N_{\mathrm{F}}}, \ldots, E_{\omega,\tau,p,n=N_{\mathrm{F}}}, H_{\omega,\tau,p,n=-N_{\mathrm{F}}}, \ldots, H_{\omega,\tau,p,n=N_{\mathrm{F}}}\right]^T, \tag{13}$$

with $-N_{\rm F} \leq p \leq N_{\rm F}$ and $\tau = 1,2$ spanning all $2\,(2N_{\rm F}+1)$ eigenvalues. Therefore, the eigenfunctions can be written as

$$E_{\omega,\tau,p}(x,t) = e^{ik_{\tau,p}x-\omega t}\sum_n E_{\omega,\tau,p,n}\, e^{i\,n(gx-\Omega t)} \tag{14}$$

and

$$H_{\omega,\tau,p}(x,t) = e^{ik_{\tau,p}x-\omega t}\sum_n H_{\omega,\tau,p,n}\, e^{i\,n(gx-\Omega t)}. \tag{15}$$

From the above, we can derive analytical expressions for the vacuum in the laboratory frame. To do so, we evaluate $\alpha_e = \alpha_m = 0$, as well as $\epsilon_m = \mu_m = 1$ in Eqs. (9-11) to obtain:

$$\mathbb{M}^{(\rm v)} = \begin{pmatrix} -ng\,\delta_{n,n'} & -\mu_0(\omega+n\Omega)\delta_{n,n'} \\ -\epsilon_0(\omega+n\Omega)\delta_{n,n'} & -ng\,\delta_{n,n'} \end{pmatrix}. \tag{16}$$

Its characteristic equation is $(k+ng)^2 - c_0^{-2}(\omega+n\Omega)^2 = 0$, such that its eigenvalues are:

$$k_n^{\pm} = -ng \pm c_0^{-1}(\omega+n\Omega). \tag{17}$$

In contrast to the eigenfunctions inside the STPhC, in vacuum we can distinguish between forward and backward modes and, furthermore, each mode will only contain one Bloch-Floquet harmonic. As such, p and n indices coincides, and the general $\tau = 1,2$ label now identifies forward and backward waves. In order to distinguish between forward and backward propagating modes, we calculate the inverse group velocity $\frac{{\rm d}k_n^{\pm}}{{\rm d}\omega} = \pm 1/c_0$ to find that it is positive for k_n^+ (forward: $k_n^+ \equiv k_{\omega,{\rm f},n}$) and negative for k_n^- (backward: $k_n^- \equiv k_{\omega,{\rm b},n}$). Therefore, the backward and forward eigenvectors are defined as

$$\begin{bmatrix} E_{\omega,{\rm f},n} \\ H_{\omega,{\rm f},n} \end{bmatrix} = \begin{bmatrix} 1 \\ \frac{-(k_{\omega,{\rm f},n}+ng)}{\mu_0(\omega+n\Omega)} \end{bmatrix}; \quad \begin{bmatrix} E_{\omega,{\rm b},n} \\ H_{\omega,{\rm b},n} \end{bmatrix} = \begin{bmatrix} 1 \\ \frac{-(k_{\omega,{\rm b},n}+ng)}{\mu_0(\omega+n\Omega)} \end{bmatrix}. \tag{18}$$

We can also obtain an eigenvalue problem for ω, which will be needed for the scattering calculation in the superluminal regime:

$$\begin{pmatrix} \mathbb{M}^{\rm EE,L}(k) & \mathbb{M}^{\rm EH,L}(k) \\ \mathbb{M}^{\rm HE,L}(k) & \mathbb{M}^{\rm HH,L}(k) \end{pmatrix} \begin{bmatrix} \mathbf{E}_k \\ \mathbf{H}_k \end{bmatrix} = \omega_k \begin{pmatrix} \mathbb{M}^{\rm EE,R}(k) & \mathbb{M}^{\rm EH,R}(k) \\ \mathbb{M}^{\rm HE,R}(k) & \mathbb{M}^{\rm HH,R}(k) \end{pmatrix} \begin{bmatrix} \mathbf{E}_k \\ \mathbf{H}_k \end{bmatrix}, \tag{19}$$

where the right matrix entries read

$$\mathbb{M}^{\rm EH,R}_{n,n'} = \mathbb{M}^{\rm HE,R}_{n,n'} = 0, \tag{20}$$

$$\mathbb{M}^{\rm EE,R}_{n,n'} = -\epsilon_0\epsilon_m[\delta_{n,n'} + \alpha_e(\delta_{n,n'+1} + \delta_{n,n'-1})], \tag{21}$$

$$\mathbb{M}^{\rm HH,R}_{n,n'} = -\mu_0\mu_m[\delta_{n,n'} + \alpha_m(\delta_{n,n'+1} + \delta_{n,n'-1})], \tag{22}$$

while the left ones are

$$\mathbb{M}^{\rm EH,L}_{n,n'} = \mathbb{M}^{\rm HE,L}_{n,n'} = (k+n\,g)\,\delta_{n,n'}, \tag{23}$$

$$\mathbb{M}^{\rm EE,L}_{n,n'} = \epsilon_0\epsilon_m(n\Omega)[\delta_{n,n'} + \alpha_e(\delta_{n,n'+1} + \delta_{n,n'-1})], \tag{24}$$

$$\mathbb{M}^{\rm HH,L}_{n,n'} = \mu_0\mu_m(n\Omega)[\delta_{n,n'} + \alpha_m(\delta_{n,n'+1} + \delta_{n,n'-1})]. \tag{25}$$

The eigenfunctions are then recovered using the same expressions as in Eqs.(14)-(15). From the above, we now derive analytical expressions for the vacuum in the laboratory frame. Again, we evaluate $\alpha_e = \alpha_m = 0$, as well as $\epsilon_m = \mu_m = 1$ in Eq. (19) to obtain:

$$\mathbb{M}^{(\rm v)} = \begin{pmatrix} -n\Omega\,\delta_{n,n'} & -\epsilon_0^{-1}(k+ng)\delta_{n,n'} \\ -\mu_0^{-1}(k+ng)\delta_{n,n'} & -n\Omega\,\delta_{n,n'} \end{pmatrix}. \tag{26}$$

Its characteristic equation is $(\omega + n\Omega)^2 - c_0^2(k+ng)^2 = 0$, such that its eigenvalues are:

$$\omega_n^{\pm} = -n\Omega \pm c_0(k+ng). \tag{27}$$

Again we find the forward modes to be $\omega_{k,\mathrm{f},n} \equiv \omega_n^+$ and the backward ones $\omega_{k,\mathrm{b},n} \equiv \omega_n^-$, with the associated eigenvectors:

$$\begin{bmatrix} E_{k,\mathrm{f},n} \\ H_{k,\mathrm{f},n} \end{bmatrix} = \begin{bmatrix} 1 \\ \frac{-(k+ng)}{\mu_0(\omega_{k,\mathrm{f},n}+n\Omega)} \end{bmatrix} ; \; \begin{bmatrix} E_{k,\mathrm{b},n} \\ H_{k,\mathrm{b},n} \end{bmatrix} = \begin{bmatrix} 1 \\ \frac{-(k+ng)}{\mu_0(\omega_{k,\mathrm{b},n}+n\Omega)} \end{bmatrix} . \tag{28}$$

B. Eigenvalue problem for the comoving frame

The magneto-electric coupling $\xi'(x')$ appearing in the constitutive matrix of Eq. (6) complicates the derivation of equivalent analytical expressions for the entries of the eigenvalue matrix. To circumvent this problem, we derive an eigenvalue problem for the comoving variables starting from the lab-frame ansatz:

$$\begin{bmatrix} E(x,t) \\ H(x,t) \end{bmatrix} = e^{i(kx-\omega t)} \sum_n \begin{bmatrix} E_n \\ H_n \end{bmatrix} e^{i\,n(gx-\Omega t)} = e^{i(k'x'-\omega' t')} \sum_n \begin{bmatrix} E_n \\ H_n \end{bmatrix} e^{i\,ng/\gamma\,x'} = \begin{bmatrix} E(x',t') \\ H(x',t') \end{bmatrix} , \tag{29}$$

where the second equality follows from the invariance of the phase under Lorentz transformations. As we can see, the Bloch-Floquet amplitudes still correspond to the lab-frame, but the field is expressed fully in the comoving frame. In the same sense, we can then derive Maxwell's equations for the lab-frame fields

$$\begin{cases} \partial_x E = \partial_t \left[\mu(x - c_g t)\, H\right] \\ \partial_x H = \partial_t \left[\epsilon(x - c_g t)\, E\right] \end{cases} , \tag{30}$$

and express them in the new coordinates as

$$\begin{cases} \partial_{x'} \left[E + c_g\, \mu(x')\, H\right] = \partial_{t'} \left[\mu(x')\, H + c_g/c_0^2 E\right] \\ \partial_{x'} \left[H + c_g\, \epsilon(x')\, E\right] = \partial_{t'} \left[\epsilon(x')\, E + c_g/c_0^2 H\right] \end{cases} . \tag{31}$$

This allows us to obtain an eigenvalue problem for (k', ω'), while avoiding the bianisotropic structure, by substituting Eq. (29) in Eq. (31):

$$\begin{pmatrix} \mathbb{M}^{\mathrm{EE,L}}(\omega') & \mathbb{M}^{\mathrm{EH,L}}(\omega') \\ \mathbb{M}^{\mathrm{HE,L}}(\omega') & \mathbb{M}^{\mathrm{HH,L}}(\omega') \end{pmatrix} \begin{bmatrix} \mathbf{E}_{\omega'} \\ \mathbf{H}_{\omega'} \end{bmatrix} = k'_{\omega'} \begin{pmatrix} \mathbb{M}^{\mathrm{EE,R}}(\omega') & \mathbb{M}^{\mathrm{EH,R}}(\omega') \\ \mathbb{M}^{\mathrm{HE,R}}(\omega') & \mathbb{M}^{\mathrm{HH,R}}(\omega') \end{pmatrix} \begin{bmatrix} \mathbf{E}_{\omega'} \\ \mathbf{H}_{\omega'} \end{bmatrix} , \tag{32}$$

where the right matrix entries read

$$\mathbb{M}^{\mathrm{EE,R}}_{n,n'} = \mathbb{M}^{\mathrm{HH,R}}_{n,n'} = -\delta_{n,n'}, \tag{33}$$

$$\mathbb{M}^{\mathrm{HE,R}}_{n,n'} = -c_g\, \epsilon_0 \epsilon_m [\delta_{n,n'} + \alpha_e(\delta_{n,n'+1} + \delta_{n,n'-1})], \tag{34}$$

$$\mathbb{M}^{\mathrm{EH,R}}_{n,n'} = -c_g\, \mu_0 \mu_m [\delta_{n,n'} + \alpha_m(\delta_{n,n'+1} + \delta_{n,n'-1})], \tag{35}$$

while the left ones are

$$\mathbb{M}^{\mathrm{EE,L}}_{n,n'} = \mathbb{M}^{\mathrm{HH,L}}_{n,n'} = (\omega' c_g/c_0^2 + ng/\gamma)\, \delta_{n,n'}, \tag{36}$$

$$\mathbb{M}^{\mathrm{HE,L}}_{n,n'} = \epsilon_0 \epsilon_m (\omega' + n\Omega/\gamma)[\delta_{n,n'} + \alpha_e(\delta_{n,n'+1} + \delta_{n,n'-1})], \tag{37}$$

$$\mathbb{M}^{\mathrm{EH,L}}_{n,n'} = \mu_0 \mu_m (\omega' + n\Omega/\gamma)[\delta_{n,n'} + \alpha_m(\delta_{n,n'+1} + \delta_{n,n'-1})]. \tag{38}$$

Once the eigenproblem is solved, we can then write the eigenfunctions for a given comoving-frame frequency ω' as

$$E_{\omega',\tau,p}(x',t') = e^{i(k'_{\omega',\tau,p}x'-\omega' t')} \sum_n E_{\omega',\tau,p,n}\, e^{i\, ng/\gamma\, x'} \tag{39}$$

and

$$H_{\omega',\tau,p}(x',t') = e^{i(k'_{\omega',\tau,p}x'-\omega' t')} \sum_n H_{\omega',\tau,p,n}\, e^{i\, ng/\gamma\, x'} . \tag{40}$$

Again, $E_{\omega',\tau,p,n}$ correspond to the first $2N_\mathrm{F}+1$ components of the eigenvector given by (τ,p), whereas $H_{\omega',\tau,p,n}$ equals the last $2N_\mathrm{F}+1$ components. However, these expressions correspond to the lab-frame fields, and we can transform them to the comoving fields with the corresponding Lorentz transformations [3]

$$\begin{bmatrix} E'(x',t') \\ H'(x',t') \end{bmatrix} = \mathbf{T}(x') \begin{bmatrix} E(x',t') \\ H(x',t') \end{bmatrix} , \tag{41}$$

with

$$\mathbf{T}(x') = \gamma \begin{pmatrix} 1 & c_g\, \mu(x') \\ c_g\, \epsilon(x') & 1 \end{pmatrix} \tag{42}$$

the transformation matrix. Thus, the comoving-frame eigenfunctions are defined as

$$E'_{\omega',\tau,p}(x',t') = e^{i(k'_{\omega',\tau,p}x'-\omega' t')} \sum_n \gamma\, \left[E_{\omega',\tau,p,n} + c_g\, \mu(x') H_{\omega',\tau,p,n}\right] e^{i\, ng/\gamma\, x'} \tag{43}$$

and

$$H'_{\omega',\tau,p}(x',t') = e^{i(k'_{\omega',\tau,p}x'-\omega' t')} \sum_n \gamma\, \left[H_{\omega',\tau,p,n} + c_g\, \epsilon(x') E_{\omega',\tau,p,n}\right] e^{i\, ng/\gamma\, x'} . \tag{44}$$

In the same manner as in the lab-frame calculation, we can derive the eigenvectors in the vacuum. To do so, we evaluate $\alpha_e = \alpha_m = 0$, as well as $\epsilon_m = \mu_m = 1$, in Eqs. (33-38) to obtain:

$$\mathbb{M}^\mathrm{R} = \begin{pmatrix} \delta_{n,n'} & c_g\mu_0\delta_{n,n'} \\ c_g\epsilon_0\delta_{n,n'} & \delta_{n,n'} \end{pmatrix} ; \;\; \mathbb{M}^\mathrm{L} = \begin{pmatrix} -(\omega' c_g/c_0^2 + ng/\gamma)\delta_{n,n'} & -\mu_0(\omega' + n\Omega/\gamma)\delta_{n,n'} \\ -\epsilon_0(\omega' + n\Omega/\gamma)\delta_{n,n'} & -(\omega' c_g/c_0^2 + ng/\gamma)\delta_{n,n'} \end{pmatrix} . \tag{45}$$

Then, rearranging Eq. (32) to have the same structure as Eq. (8), we obtain the eigenvalue matrix in the comoving frame by defining $\mathbb{M}^{(\mathrm{v})} = (\mathbb{M}^\mathrm{R})^{-1}\, \mathbb{M}^\mathrm{L}$, which corresponds to

$$\mathbb{M}^{(\mathrm{v})} = \begin{pmatrix} -ng/\gamma\, \delta_{n,n'} & -\mu_0\omega'\delta_{n,n'} \\ -\epsilon_0\omega'\delta_{n,n'} & -ng/\gamma\, \delta_{n,n'} \end{pmatrix} . \tag{46}$$

Its characteristic equation is then $(k' + ng/\gamma)^2 - (\omega'/c_0)^2 = 0$, so the comoving-frame eigenvalues of free space are:

$$k'^{\pm}_n = -ng/\gamma \pm \omega'/c_0 \tag{47}$$

Since we want to distinguish between forward and backward modes, we calculate the inverse group velocity. We find the same as in the lab-frame case: it is positive for k'^{+}_n (forward: $k'^{+}_n \equiv k'_{\omega',\mathrm{f},n}$) and negative for k'^{-}_n (backward: $k'^{-}_n \equiv k'_{\omega',\mathrm{b},n}$). Therefore, the backward and forward eigenvectors are defined as

$$\begin{bmatrix} E_{\omega',\mathrm{f},n} \\ H_{\omega',\mathrm{f},n} \end{bmatrix} = \begin{bmatrix} 1 \\ \frac{-(k'_{\omega',\mathrm{f},n}+ng/\gamma)}{\mu_0\omega'} \end{bmatrix} = \frac{1}{\sqrt{1+\eta^{-2}}} \begin{bmatrix} 1 \\ -\eta^{-1} \end{bmatrix} ; \;\; \begin{bmatrix} E_{\omega',\mathrm{b},n} \\ H_{\omega',\mathrm{b},n} \end{bmatrix} = \begin{bmatrix} 1 \\ \frac{-(k'_{\omega',\mathrm{b},n}+ng/\gamma)}{\mu_0\omega'} \end{bmatrix} = \frac{1}{\sqrt{1+\eta^{-2}}} \begin{bmatrix} 1 \\ \eta^{-1} \end{bmatrix} , \tag{48}$$

where $\eta^2 = \mu_0/\epsilon_0$ is the wave impedance in vacuum.

On the other hand, we can also derive an eigenvalue problem with ω' as eigenvalue

$$\begin{pmatrix} \mathbb{M}^{\mathrm{EE,L}}(k') & \mathbb{M}^{\mathrm{EH,L}}(k') \\ \mathbb{M}^{\mathrm{HE,L}}(k') & \mathbb{M}^{\mathrm{HH,L}}(k') \end{pmatrix} \begin{bmatrix} \mathbf{E}_{k'} \\ \mathbf{H}_{k'} \end{bmatrix} = \omega'_{k'} \begin{pmatrix} \mathbb{M}^{\mathrm{EE,R}}(k') & \mathbb{M}^{\mathrm{EH,R}}(k') \\ \mathbb{M}^{\mathrm{HE,R}}(k') & \mathbb{M}^{\mathrm{HH,R}}(k') \end{pmatrix} \begin{bmatrix} \mathbf{E}_{k'} \\ \mathbf{H}_{k'} \end{bmatrix}, \tag{49}$$

where the right matrix entries read

$$\mathbb{M}^{\mathrm{EH,R}}_{n,n'} = \mathbb{M}^{\mathrm{HE,R}}_{n,n'} = -c_g/c_0^2\, \delta_{n,n'}, \tag{50}$$

$$\mathbb{M}^{\mathrm{EE,R}}_{n,n'} = -\epsilon_0\epsilon_m[\delta_{n,n'} + \alpha_e(\delta_{n,n'+1} + \delta_{n,n'-1})], \tag{51}$$

$$\mathbb{M}^{\mathrm{HH,R}}_{n,n'} = -\mu_0\mu_m[\delta_{n,n'} + \alpha_m(\delta_{n,n'+1} + \delta_{n,n'-1})], \tag{52}$$

while the left ones are

$$\mathbb{M}^{\mathrm{EH,L}}_{n,n'} = \mathbb{M}^{\mathrm{HE,L}}_{n,n'} = (k' + n\, g/\gamma)\, \delta_{n,n'}, \tag{53}$$

$$\mathbb{M}^{\mathrm{EE,L}}_{n,n'} = c_g\, \epsilon_0\epsilon_m(k' + n\, g/\gamma)[\delta_{n,n'} + \alpha_e(\delta_{n,n'+1} + \delta_{n,n'-1})], \tag{54}$$

$$\mathbb{M}^{\mathrm{HH,L}}_{n,n'} = c_g\, \mu_0\mu_m(k' + n\, g/\gamma)[\delta_{n,n'} + \alpha_m(\delta_{n,n'+1} + \delta_{n,n'-1})]. \tag{55}$$

Once solved, we can recover the comoving-frame eigenfunctions as

$$E'_{k',m}(x',t') = e^{i(k'x' - \omega'_{k',m}t')} \underbrace{\sum_n \gamma\, [E_{k',m,n} + c_g\, \mu(x') H_{k',m,n}]\, e^{i\, ng/\gamma\, x'}}_{u'^{E}_{k',m}(x')} \tag{56}$$

and

$$H'_{k',m}(x',t') = e^{i(k'x' - \omega'_{k',m}t')} \underbrace{\sum_n \gamma\, [H_{k',m,n} + c_g\, \epsilon(x') E_{k',m,n}]\, e^{i\, ng/\gamma\, x'}}_{u'^{H}_{k',m}(x')}. \tag{57}$$

These expressions are equivalent to Eqs. (43)-(44), however, they are now labeled by k' and the band index m.

II. SCATTERING MATRIX AND TRANSMISSION SPECTRUM

Once the eigenvalue problem for the STPhC is solved, we can compute the scattering matrix of a finite system composed of two slabs separated by either spatial or ST boundaries. The boundary conditions differ depending on the type of interface: a static boundary conserves the laboratory-frame frequency ω, whereas a ST boundary conserves the comoving-frame frequency ω'. Consequently, for each case we must select the eigenfunctions of the STPhC that possess a well-defined ω or ω', such that we use those obtained from Eq. (8) for spatial boundaries and from Eq. (32) for spatiotemporal ones.

A. Spatiotemporal Boundary

We start by defining the scattering matrix of two STPhC slabs truncated with ST interfaces. To do so, we need to enforce the continuity of the comoving-frame E'_z and H'_y fields at each interface, which are easily defined as $x' = 0, N\gamma a, 2N\gamma a$, where N is the number of ST unit cells (defined as $x' \in [0, \gamma a]$) used to construct each slab. Making use of the field decomposition, we can write the matching conditions using a superposition of the eigenvectors as

$$\begin{bmatrix} \mathbf{E}'_{\omega'} \\ \mathbf{H}'_{\omega'} \end{bmatrix}^{(\mathrm{m})} = \mathcal{M}'^{(\mathrm{m})}_{\omega'}\, \mathbf{e}^{(\mathrm{m})}_{\omega'}; \quad \begin{bmatrix} \mathbf{E}'_{\omega'} \\ \mathbf{H}'_{\omega'} \end{bmatrix}^{(\mathrm{in/out})}_{\mathrm{f/b}} = \mathcal{M}'^{(\mathrm{v})}_{\omega',\mathrm{f/b}}\, \mathbf{e}^{(\mathrm{in/out})}_{\omega',\mathrm{f/b}}, \tag{58}$$

with

$$\mathcal{M}'^{(\mathrm{m})}_{\omega'} = \mathbf{T}^{(\mathrm{m})}\mathcal{M}^{(\mathrm{m})}_{\omega'}; \quad \mathcal{M}'^{(\mathrm{v})}_{\omega',\mathrm{f/b}} = \mathbf{T}^{(\mathrm{v})}\mathcal{M}^{(\mathrm{v})}_{\omega',\mathrm{f/b}}, \tag{59}$$

where $\mathcal{M}^{(\mathrm{m})}_{\omega'}$ is a $2\,(2N_\mathrm{F}+1)\times 2\,(2N_\mathrm{F}+1)$ matrix equivalent to the one defined in Eq. (12) for the eigenvectors containing the amplitudes given by Eq. (32), and $\mathbf{e}^{(\mathrm{m})}_{\omega'}$ a $2\,(2N_\mathrm{F}+1)$ vector containing the scattering amplitudes of each eigenvector. $\mathcal{M}'^{(\mathrm{v})}_{\omega',\mathrm{f/b}}$ correspond to $2\,(2N_\mathrm{F}+1)\times(2N_\mathrm{F}+1)$ matrices containing the forward and backward vacuum modes given by Eq. (103), with $\mathbf{e}^{(\mathrm{in/out})}_{\omega',\mathrm{f/b}}$ the amplitudes of the input and output waves in both directions of propagation. Finally, the $\mathbf{T}^{(\mathrm{v})}$ and $\mathbf{T}^{(\mathrm{m})}$ matrices correspond to the transformation matrix of Eq. (42) evaluated at the interfaces for the unmodulated and modulated cases, respectively. The periodicity of the modulation ensures that at the boundaries the matrices read

$$\mathbf{T}^{(\mathrm{v})} = \gamma\begin{pmatrix} \mathbb{1} & c_g\mu_0\mathbb{1} \\ c_g\epsilon_0\mathbb{1} & \mathbb{1}\end{pmatrix}; \quad \mathbf{T}^{(\mathrm{m})} = \gamma\begin{pmatrix} \mathbb{1} & c_g\mu_0\mu_m(1+2\alpha_m)\mathbb{1} \\ c_g\epsilon_0\epsilon_m(1+2\alpha_e)\mathbb{1} & \mathbb{1}\end{pmatrix}. \tag{60}$$

We now enforce the boundary conditions at each interface:

- 1st interface:

$$\begin{bmatrix}\mathbf{E}'_{\omega'}\\ \mathbf{H}'_{\omega'}\end{bmatrix}^{(\mathrm{in}),1}_{\mathrm{f}} + \begin{bmatrix}\mathbf{E}'_{\omega'}\\ \mathbf{H}'_{\omega'}\end{bmatrix}^{(\mathrm{out}),1}_{\mathrm{b}} = \begin{bmatrix}\mathbf{E}'_{\omega'}\\ \mathbf{H}'_{\omega'}\end{bmatrix}^{(\mathrm{m}),1} \to \mathcal{M}'^{(\mathrm{v})}_{\omega',\mathrm{f}}\,\mathbf{e}^{(\mathrm{in}),1}_{\omega',\mathrm{f}} + \mathcal{M}'^{(\mathrm{v})}_{\omega',\mathrm{b}}\,\mathbf{e}^{(\mathrm{out}),1}_{\omega',\mathrm{b}} = \mathcal{M}'^{(\mathrm{m})}_{\omega'}\,\mathbf{e}^{(\mathrm{m}),1}_{\omega'}. \tag{61}$$

- 2nd interface:

$$\begin{bmatrix}\mathbf{E}'_{\omega'}\\ \mathbf{H}'_{\omega'}\end{bmatrix}^{(\mathrm{m}),2} = \begin{bmatrix}\mathbf{E}'_{\omega'}\\ \mathbf{H}'_{\omega'}\end{bmatrix}^{(\tilde{\mathrm{m}}),2} \to \mathcal{M}'^{(\mathrm{m})}_{\omega'}\,\mathbf{e}^{(\mathrm{m}),2}_{\omega'} = \mathcal{M}'^{(\mathrm{m})}_{\omega'}\,\mathbf{P}'\,\mathbf{e}^{(\mathrm{m}),1}_{\omega'} = \mathcal{M}'^{(\tilde{\mathrm{m}})}_{\omega'}\,\mathbf{e}^{(\tilde{\mathrm{m}}),2}_{\omega'}. \tag{62}$$

- 3rd interface:

$$\begin{bmatrix}\mathbf{E}'_{\omega'}\\ \mathbf{H}'_{\omega'}\end{bmatrix}^{(\tilde{\mathrm{m}}),3} = \begin{bmatrix}\mathbf{E}'_{\omega'}\\ \mathbf{H}'_{\omega'}\end{bmatrix}^{(\mathrm{out}),3}_{\mathrm{f}} + \begin{bmatrix}\mathbf{E}'_{\omega'}\\ \mathbf{H}'_{\omega'}\end{bmatrix}^{(\mathrm{in}),3}_{\mathrm{b}} \to \mathcal{M}'^{(\tilde{\mathrm{m}})}_{\omega'}\,\mathbf{e}^{(\tilde{\mathrm{m}}),3}_{\omega'} = \mathcal{M}'^{(\tilde{\mathrm{m}})}_{\omega'}\,\tilde{\mathbf{P}}'\,\mathbf{e}^{(\tilde{\mathrm{m}}),2}_{\omega'} = \mathcal{M}'^{(\mathrm{v})}_{\omega',\mathrm{f}}\,\mathbf{e}^{(\mathrm{out}),3}_{\omega',\mathrm{f}} + \mathcal{M}'^{(\mathrm{v})}_{\omega',\mathrm{b}}\,\mathbf{e}^{(\mathrm{in}),3}_{\omega',\mathrm{b}}. \tag{63}$$

$\mathbf{P}'$ corresponds to the diagonal matrix containing the phase acquired by each eigenvector between surfaces $P'_{jj} = \exp(i\,k'_j D')$, where $D' = N\gamma a$ is the width of each slab and k'_j the eigenvalues of the STPhC. The matrix containing the eigenvectors of the second slab is represented with ($\tilde{\mathrm{m}}$), and its corresponding phase matrix as $\tilde{\mathbf{P}}'$. Now, multiplying Eq. (63) by $\mathcal{M}'^{(\tilde{\mathrm{m}})}_{\omega'}(\mathcal{M}'^{(\tilde{\mathrm{m}})}_{\omega'}\tilde{\mathbf{P}}')^{-1}$ we obtain:

$$\mathcal{M}'^{(\tilde{\mathrm{m}})}_{\omega'}(\mathcal{M}'^{(\tilde{\mathrm{m}})}_{\omega'}\tilde{\mathbf{P}}')^{-1}\left(\mathcal{M}'^{(\mathrm{v})}_{\omega',\mathrm{f}}\,\mathbf{e}^{(\mathrm{out}),3}_{\omega',\mathrm{f}} + \mathcal{M}'^{(\mathrm{v})}_{\omega',\mathrm{b}}\,\mathbf{e}^{(\mathrm{in}),3}_{\omega',\mathrm{b}}\right) = \mathcal{M}'^{(\tilde{\mathrm{m}})}_{\omega'}\,\mathbf{e}^{(\tilde{\mathrm{m}})}_{\omega,(2)} = \mathcal{M}'^{(\mathrm{m})}_{\omega'}\,\mathbf{P}'\,\mathbf{e}^{m}_{\omega,(1)}, \tag{64}$$

where we have used Eq. (62) for the second equality. Then, multiplying again the previous equation by $\mathcal{M}'^{(\mathrm{m})}_{\omega'}(\mathcal{M}'^{(\mathrm{m})}_{\omega'}\mathbf{P}')^{-1}$ and using Eq. (61) leads to:

$$\mathcal{M}'^{(\mathrm{m})}_{\omega'}(\mathcal{M}'^{(\mathrm{m})}_{\omega'}\mathbf{P}')^{-1}\,\mathcal{M}'^{(\tilde{\mathrm{m}})}_{\omega'}(\mathcal{M}'^{(\tilde{\mathrm{m}})}_{\omega'}\tilde{\mathbf{P}}')^{-1}\left(\mathcal{M}'^{(\mathrm{v})}_{\omega',\mathrm{f}}\,\mathbf{e}^{(\mathrm{out}),3}_{\omega',\mathrm{f}} + \mathcal{M}'^{(\mathrm{v})}_{\omega',\mathrm{b}}\,\mathbf{e}^{(\mathrm{in}),3}_{\omega',\mathrm{b}}\right) = \mathcal{M}'^{(\mathrm{v})}_{\omega',\mathrm{f}}\,\mathbf{e}^{(\mathrm{in}),1}_{\omega',\mathrm{f}} + \mathcal{M}'^{(\mathrm{v})}_{\omega',\mathrm{b}}\,\mathbf{e}^{(\mathrm{out}),1}_{\omega',\mathrm{b}}. \tag{65}$$

Finally, after rearranging and defining $\mathbf{N}_{\omega'} = \mathcal{M}'^{(\mathrm{m})}_{\omega'}(\mathcal{M}'^{(\mathrm{m})}_{\omega'}\mathbf{P}')^{-1}\,\mathcal{M}'^{(\tilde{\mathrm{m}})}_{\omega'}(\mathcal{M}'^{(\tilde{\mathrm{m}})}_{\omega'}\tilde{\mathbf{P}}')^{-1}$ we arrive at an expression relating the output amplitudes $\mathbf{e}^{(\mathrm{out}),3}_{\omega',\mathrm{f}}$ and $\mathbf{e}^{(\mathrm{out}),1}_{\omega',\mathrm{b}}$ to the input ones $\mathbf{e}^{(\mathrm{in}),1}_{\omega',\mathrm{f}}$ and $\mathbf{e}^{(\mathrm{in}),3}_{\omega',\mathrm{b}}$:

$$\mathbf{N}_{\omega'}\mathcal{M}'^{(\mathrm{v})}_{\omega',\mathrm{f}}\,\mathbf{e}^{(\mathrm{out}),3}_{\omega',\mathrm{f}} - \mathcal{M}'^{(\mathrm{v})}_{\omega',\mathrm{b}}\,\mathbf{e}^{(\mathrm{out}),1}_{\omega',\mathrm{b}} = \mathcal{M}'^{(\mathrm{v})}_{\omega',\mathrm{f}}\,\mathbf{e}^{(\mathrm{in}),1}_{\omega',\mathrm{f}} - \mathbf{N}_{\omega'}\mathcal{M}'^{(\mathrm{v})}_{\omega',\mathrm{b}}\,\mathbf{e}^{(\mathrm{in}),3}_{\omega',\mathrm{b}}. \tag{66}$$

We can express the previous equation in vector form:

$$\underbrace{\left[\mathbf{N}_{\omega'}\mathcal{M}'^{(\mathrm{v})}_{\omega',\mathrm{f}}\,,\, -\mathcal{M}'^{(\mathrm{v})}_{\omega',\mathrm{b}}\right]}_{\mathbf{A}}\begin{bmatrix}\mathbf{e}^{(\mathrm{out}),3}_{\omega',\mathrm{f}}\\ \mathbf{e}^{(\mathrm{out}),1}_{\omega',\mathrm{b}}\end{bmatrix} = \underbrace{\left[\mathcal{M}'^{(\mathrm{v})}_{\omega',\mathrm{f}}\,,\, -\mathbf{N}_{\omega'}\mathcal{M}'^{(\mathrm{v})}_{\omega',\mathrm{b}}\right]}_{\mathbf{B}}\begin{bmatrix}\mathbf{e}^{(\mathrm{in}),1}_{\omega',\mathrm{f}}\\ \mathbf{e}^{(\mathrm{in}),3}_{\omega',\mathrm{b}}\end{bmatrix}, \tag{67}$$

where we have defined the $2 \cdot (2N_{\mathrm{F}}+1) \times 2 \cdot (2N_{\mathrm{F}}+1)$ matrices $\mathbf{A}$ and $\mathbf{B}$. It is now easy to define our scattering matrix $\mathbf{S}_{\omega'}$ as

$$\begin{bmatrix} \mathbf{e}^{(\mathrm{out}),3}_{\omega',\mathrm{f}} \\ \mathbf{e}^{(\mathrm{out}),1}_{\omega',\mathrm{b}} \end{bmatrix} = \mathbf{S}_{\omega'} \begin{bmatrix} \mathbf{e}^{(\mathrm{in}),1}_{\omega',\mathrm{f}} \\ \mathbf{e}^{(\mathrm{in}),3}_{\omega',\mathrm{b}} \end{bmatrix}, \tag{68}$$

with $\mathbf{S}_{\omega'} = \mathbf{A}^{-1}\,\mathbf{B}$.

Furthermore, since we want to see the existence of interface states between slabs, we need to extract the vector $\mathbf{e}^{(\mathrm{m}),i}_{\omega}$ at each interface i in terms of the weights of the input fields. To derive their expression, we make use of the previous mode-matching equations (61) and substitute $\mathbf{e}^{(\mathrm{out}),1}_{\omega',\mathrm{b}}$ expressed in terms of the incident fields: $\mathbf{e}^{(\mathrm{out}),1}_{\omega',\mathrm{b}} = \mathbf{S}^{21}_{\omega'}\,\mathbf{e}^{(\mathrm{in}),1}_{\omega',\mathrm{f}} + \mathbf{S}^{22}_{\omega'}\,\mathbf{e}^{(\mathrm{in}),3}_{\omega',\mathrm{b}}$. We would then have:

$$\mathcal{M}'^{(\mathrm{m})}_{\omega'}\,\mathbf{e}^{(\mathrm{m}),1}_{\omega'} = (\mathcal{M}'^{(\mathrm{v})}_{\omega',\mathrm{f}} + \mathcal{M}'^{(\mathrm{v})}_{\omega',\mathrm{b}}\,\mathbf{S}^{21}_{\omega'})\,\mathbf{e}^{(\mathrm{in}),1}_{\omega',\mathrm{f}} + \mathcal{M}'^{(\mathrm{v})}_{\omega',\mathrm{b}}\,\mathbf{S}^{22}_{\omega'}\mathbf{e}^{(\mathrm{in}),3}_{\omega',\mathrm{b}}, \tag{69}$$

$$\mathbf{e}^{(\mathrm{m}),1}_{\omega'} = (\mathcal{M}'^{(\mathrm{m})}_{\omega'})^{-1}\,\underbrace{\left[\mathcal{M}'^{(\mathrm{v})}_{\omega',\mathrm{f}} + \mathcal{M}'^{(\mathrm{v})}_{\omega',\mathrm{b}}\,\mathbf{S}^{21}_{\omega'}\,,\,\mathcal{M}'^{(\mathrm{v})}_{\omega',\mathrm{b}}\,\mathbf{S}^{22}_{\omega'}\right]}_{\mathbf{C}} \begin{bmatrix} \mathbf{e}^{(\mathrm{in}),1}_{\omega',\mathrm{f}} \\ \mathbf{e}^{(\mathrm{in}),3}_{\omega',\mathrm{b}} \end{bmatrix}. \tag{70}$$

Once we have the coefficients for the first slab, we can repeat the same process for the second one, taking into account that the relevant mode-matching equation is now Eq. (62), such that:

$$\mathbf{e}^{(\tilde{\mathrm{m}}),2}_{\omega'} = (\mathcal{M}'^{(\tilde{\mathrm{m}})}_{\omega})^{-1}\,\mathcal{M}'^{(\mathrm{m})}_{\omega}\,\mathbf{P}'\,(\mathcal{M}'^{(\mathrm{m})}_{\omega})^{-1}\,\mathbf{C} \begin{bmatrix} \mathbf{e}^{(\mathrm{in}),1}_{\omega',\mathrm{f}} \\ \mathbf{e}^{(\mathrm{in}),3}_{\omega',\mathrm{b}} \end{bmatrix}. \tag{71}$$

Finally, to obtain the EM fields we only need to substitute the previous magnitudes into the following equation for the fields inside the material:

$$\begin{aligned} \begin{bmatrix} E'(x',t') \\ H'(x',t') \end{bmatrix}^{(\mathrm{m})} &= \mathbf{T}(x') \sum_{\tau=1,2} \sum_{p=-N_{\mathrm{F}}}^{N_{\mathrm{F}}} e^{(\mathrm{m})}_{\omega',\tau,p} \begin{bmatrix} E_{\omega',\tau,p}(x',t') \\ H_{\omega',\tau,p}(x',t') \end{bmatrix}^{(\mathrm{m})} = \\ &= \mathbf{T}(x') \sum_{\tau=1,2} \sum_{p=-N_{\mathrm{F}}}^{N_{\mathrm{F}}} e^{(\mathrm{m})}_{\omega',\tau,p} \sum_{n=-N_{\mathrm{F}}}^{N_{\mathrm{F}}} \begin{bmatrix} E_{\omega',\tau,p,n} \\ H_{\omega',\tau,p,n} \end{bmatrix}^{(\mathrm{m})} e^{i(k'_{\omega',\tau,p}+ng/\gamma)x' - i\omega' t'}, \end{aligned} \tag{72}$$

and into this expression for the fields in vacuum:

$$\begin{aligned} \begin{bmatrix} E'(x',t') \\ H'(x',t') \end{bmatrix}^{(\mathrm{in/out})}_{\mathrm{f/b}} &= \mathbf{T}^{(\mathrm{v})} \sum_{n=-N_{\mathrm{F}}}^{N_{\mathrm{F}}} e^{(\mathrm{in/out})}_{\omega',\mathrm{f/b},n} \begin{bmatrix} E_{\omega',\mathrm{f/b},n}(x',t') \\ H_{\omega',\mathrm{f/b},n}(x',t') \end{bmatrix}^{(\mathrm{in/out})} = \\ &= \mathbf{T}^{(\mathrm{v})} \sum_{n=-N_{\mathrm{F}}}^{N_{\mathrm{F}}} e^{(\mathrm{in/out})}_{\omega',\mathrm{f/b},n} \begin{bmatrix} E_{\omega',\mathrm{f/b},n} \\ H_{\omega',\mathrm{f/b},n} \end{bmatrix}^{(\mathrm{in/out})} e^{i(k'_{\omega',\mathrm{f/b},n}+ng/\gamma)x' - i\omega' t'}. \end{aligned} \tag{73}$$

Lastly, we calculate the transmission spectrum in the lab-frame obtained by exciting the composite system from the left side with a plane wave of frequency ω_0. To do so, we transform the incident frequency to the comoving frame, since it corresponds to the conserved quantity, and then we use the scattering matrix defined in Eq. (68) to calculate the transmitted fields as seen in the lab-frame. Once the fields are obtained, we can then evaluate the time-averaged Poynting vector in the x' direction, defined as:

$$P^{(\mathrm{out})}_{\omega',\mathrm{f}}(x') = -E^{(\mathrm{out})}_{\omega',\mathrm{f}}(x')\left(H^{(\mathrm{out})}_{\omega',\mathrm{f}}(x')\right)^* = -\sum_{n,n'} e^{(\mathrm{out})}_{\omega',\mathrm{f},n}\,e^{(\mathrm{out})\,*}_{\omega',\mathrm{f},n'}\,E^{(\mathrm{out})}_{\omega',\mathrm{f},n}\,H^{(\mathrm{out})\,*}_{\omega',\mathrm{f},n'}\,e^{i\left(k'_{\omega',\mathrm{f},n} - k'^{*}_{\omega',\mathrm{f},n'} + (n-n')g/\gamma\right)x'}. \tag{74}$$

Then, normalizing the previous expression by the Poynting vector of the incident wave, and evaluating at the third interface $x' = 2\gamma N a$, we obtain the transmittance as

$$T(\omega') = \frac{P^{(\mathrm{out})}_{\omega',\mathrm{f}}(x' = 2\gamma N a)}{P^{(\mathrm{in})}_{\omega',\mathrm{f}}(x' = 0)} = \frac{\sum_{n,n'} e^{(\mathrm{out})}_{\omega',\mathrm{f},n}\,e^{(\mathrm{out})\,*}_{\omega',\mathrm{f},n'}\,E^{(\mathrm{out})}_{\omega',\mathrm{f},n}\,H^{(\mathrm{out})\,*}_{\omega',\mathrm{f},n'}\,e^{i\left(k'_{\omega',\mathrm{f},n} - k'^{*}_{\omega',\mathrm{f},n'}\right)(2\gamma N a)}}{E^{(\mathrm{in})}_{\omega',\mathrm{f},0}H^{(\mathrm{in})\,*}_{\omega',\mathrm{f},0}}. \tag{75}$$

B. Spatial Boundary

The procedure to calculate the scattering matrix for two slabs truncated purely in space closely follows that described in the previous section. The main differences lie in the conserved quantity, which is now the lab-frame frequency, and in the boundary conditions enforcing the continuity of the lab-frame electromagnetic fields. These modifications can be taken into account by substituting the eigenvector matrices of both the vacuum and the modulated medium with those derived in Sec. I A, where ω rather than ω' is well defined, as well as changing the transformation matrices in Eq. (106) to the identity. In this way, Eqs. (61)-(63) naturally enforce the continuity of $[E, H]^{\mathrm{T}}$ instead of $[E', H']^{\mathrm{T}}$.

Finally, due to the intrinsic temporal dependence of the eigenfunctions defined in Eq. (14)-(15), the time averaged Poynting vector still has an explicit temporal dependence

$$P^{(\mathrm{out})}_{\omega,\mathrm{f}}(x,t) = -E^{(\mathrm{out})}_{\omega,\mathrm{f}}(x,t)\,\left(H^{(\mathrm{out})}_{\omega,\mathrm{f}}(x,t)\right)^* = \\ = -\sum_{n,n'} e^{(\mathrm{out})}_{\omega,\mathrm{f},n}\, e^{(\mathrm{out})\,*}_{\omega,\mathrm{f},n'}\, E^{(\mathrm{out})}_{\omega,\mathrm{f},n,n}\, H^{(\mathrm{out})\,*}_{\omega,\mathrm{f},n',n'} e^{i\left(k_{\omega,\mathrm{f},n}-k^*_{\omega,\mathrm{f},n'}+(n-n')g\right)x-i(n-n')\Omega t}. \tag{76}$$

We next integrate the time averaged Poynting vector over one modulation period $T = 2\pi/\Omega$

$$\langle P^{(\mathrm{out})}_{\omega,\mathrm{f}}\rangle = \frac{1}{T}\int_0^T P^{(\mathrm{out})}_{\omega,\mathrm{f}} = -\sum_{n,n'} \delta_{n,n'}\, e^{(\mathrm{out})}_{\omega,\mathrm{f},n}\, e^{(\mathrm{out})\,*}_{\omega,\mathrm{f},n'}\, E^{(\mathrm{out})}_{\omega,\mathrm{f},n}\, H^{(\mathrm{out})\,*}_{\omega,\mathrm{f},n'}\, e^{i\left(k_{\omega,\mathrm{f},n}-k^*_{\omega,\mathrm{f},n'}+(n-n')g\right)x}, \tag{77}$$

and finally we arrive at the transmittance for the spatial boundary case

$$T(\omega) = \frac{\langle P^{(\mathrm{out})}_{\omega,\mathrm{f}}(x=2Na)\rangle}{\langle P^{(\mathrm{in})}_{\omega,\mathrm{f}}(x=0)\rangle} = \frac{\sum_n |e^{(\mathrm{out})}_{\omega,\mathrm{f},n}|^2 E^{(\mathrm{out})}_{\omega,\mathrm{f},n}\, H^{(\mathrm{out})\,*}_{\omega,\mathrm{f},n}}{E^{(\mathrm{in})}_{\omega,\mathrm{f},0} H^{(\mathrm{in})\,*}_{\omega,\mathrm{f},0}}. \tag{78}$$

III. SPATIOTEMPORAL ZAK PHASE

As discussed in the main text, the Hermiticity of the constitutive matrix, together with the presence of $\mathcal{P}'\mathcal{T}'$ symmetry, allows us to define a Hermitian form of the spatiotemporal Zak phase. Under these conditions, left and right eigenfunctions can be related, which converts the biorthogonal Berry connection into a conventional one defined through a weighted inner product. To demonstrate this, we derive a general eigenvalue problem from Eq. (6) by considering monochromatic fields such that

$$\hat{\mathbb{L}}'\mathbf{\Psi}'_{\mathrm{R}} = \omega'\,\hat{\mathbf{M}}'\mathbf{\Psi}'_{\mathrm{R}}, \tag{79}$$

where $\hat{\mathbb{L}}' = i\hat{\mathbf{L}}'$. As we can see, both $\hat{\mathbb{L}}'$ and $\hat{\mathbf{M}}'(x')$ operators are Hermitian. However, if we write Eq. (79) as

$$\hat{\mathbf{H}}_{\mathrm{R}}\mathbf{\Psi}'_{\mathrm{R}} = \left(\hat{\mathbf{M}}'\right)^{-1}\hat{\mathbb{L}}'\mathbf{\Psi}'_{\mathrm{R}} = \omega'\,\mathbf{\Psi}'_{\mathrm{R}}, \tag{80}$$

we clearly see that the operator $\hat{\mathbf{H}}_{\mathrm{R}}$ is not Hermitian, since the previous operators do not commute. One can prove that the adjoint operator of $\hat{\mathbf{H}}_{\mathrm{R}}$ is [4]

$$\hat{\mathbf{H}}_{\mathrm{L}} = \hat{\mathbb{L}}'\left(\hat{\mathbf{M}}'\right)^{-1} = \left(\hat{\mathbf{H}}_{\mathrm{R}}\right)^{\dagger}, \text{with} \quad \hat{\mathbf{H}}_{\mathrm{L}}\mathbf{\Psi}'_{\mathrm{L}} = (\omega')^*\,\mathbf{\Psi}'_{\mathrm{L}}. \tag{81}$$

Now, writing the adjoint operator as $\hat{\mathbf{H}}_{\mathrm{L}} = \hat{\mathbf{M}}'\left(\hat{\mathbf{M}}'\right)^{-1}\hat{\mathbb{L}}'\left(\hat{\mathbf{M}}'\right)^{-1}$, we arrive at the following expression

$$\hat{\mathbf{M}}'\underbrace{\left(\hat{\mathbf{M}}'\right)^{-1}\hat{\mathbb{L}}'}_{\hat{\mathbf{H}}_{\mathrm{R}}}\left(\hat{\mathbf{M}}'\right)^{-1}\mathbf{\Psi}'_{\mathrm{L}} = (\omega')^*\,\mathbf{\Psi}'_{\mathrm{L}} \longrightarrow \hat{\mathbf{H}}_{\mathrm{R}}\left(\hat{\mathbf{M}}'\right)^{-1}\mathbf{\Psi}'_{\mathrm{L}} = (\omega')^*\,\left(\hat{\mathbf{M}}'\right)^{-1}\mathbf{\Psi}'_{\mathrm{L}}. \tag{82}$$

Considering in this section the subluminal regime, we know from the conservation of $\mathcal{P}'\mathcal{T}'$-symmetry that the frequency spectrum is real $(\omega')^* = \omega'$, which means

$$\mathbf{\Psi}'_{\mathrm{R}} = \left(\hat{\mathbf{M}}'\right)^{-1}\mathbf{\Psi}'_{\mathrm{L}}, \tag{83}$$

such that the inner product of left and right eigenfunctions becomes

$$\langle \mathbf{\Psi}'_{\mathrm{L}} | \mathbf{\Psi}'_{\mathrm{R}} \rangle = \int_{\mathrm{UC}} dx' \left(\mathbf{\Psi}'_{\mathrm{L}}\right)^{\dagger} \mathbf{\Psi}'_{\mathrm{R}} = \int_{\mathrm{UC}} dx' \left(\hat{\mathbf{M}}' \mathbf{\Psi}'_{\mathrm{R}}\right)^{\dagger} \mathbf{\Psi}'_{\mathrm{R}} = \int_{\mathrm{UC}} dx' \left(\mathbf{\Psi}'_{\mathrm{R}}\right)^{\dagger} \hat{\mathbf{M}}' \mathbf{\Psi}'_{\mathrm{R}}. \tag{84}$$

This relation allows us to write the Zak phase defined with a biorthogonal Berry connection as

$$\theta_m^{\mathrm{ST}} = i \int_{-g/2\gamma}^{+g/2\gamma} dk' \, \langle \mathbf{u}'_{\mathrm{L},k',m} | \partial_{k'} \mathbf{u}'_{\mathrm{R},k',m} \rangle = i \int_{-g/2\gamma}^{+g/2\gamma} dk' \, \langle \mathbf{u}'_{\mathrm{R},k',m} | \hat{\mathbf{M}}' \partial_{k'} \mathbf{u}'_{\mathrm{R},k',m} \rangle \,, \tag{85}$$

which ends with the same structure as a conventional Berry connection defined with a weighted scalar product, as mentioned in the main text.

To obtain numerically the ST Zak phase, we make use of the eigenfunctions defined in Eqs. (56)-(57) and the discretized Zak phase definition:

$$\theta_m^{\mathrm{ST}} = -\mathrm{Im} \, \log \prod_i \langle \mathbf{u}'_{k'_i,m} | \mathbf{u}'_{k'_i+1,m} \rangle_{\hat{\mathbf{M}}} \,, \tag{86}$$

where $\langle \cdot | \cdot \rangle_{\hat{\mathbf{M}}}$ correspond to the weighted scalar product from Eq. (17) of the main text, and we enforce the periodic gauge condition $\mathbf{u}'_{k'=g/2\gamma,m}(x') = e^{-ig/\gamma x'} \mathbf{u}'_{k'=-g/2\gamma,m}(x')$.

Studying these scalar products along the comoving-frame BZ, we find that they are always real, implying that the Zak phase is determined by the overall sign of the product in Eq. (86). The sign changes occur when crossing the band gaps and at $k' = \omega' = 0$. Importantly, the scalar products involving the $k' = 0$ eigenfunctions for the first band exhibit inconsistent sign changes depending on the specific parameters of the STPhC. Although the two phases cannot be unambiguously assigned to $\alpha > 0$ or $\alpha < 0$, they remain clearly distinguished by distinct ST Zak phase values, which is enough to predict interface states. This ambiguity arises because, while the electric and magnetic eigenfunctions possess opposite parity, they become constant at $k' = \omega' = 0$, rendering their parity ill-defined and leading to inconsistent phase assignments.

IV. BAND CROSSING POSITION

In Fig. S1, we show how to infer the resonance frequency of the interface states from the position in the band diagram where the $(0, -1)$ Floquet bands cross, and considering the conserved quantity in the scattering process. We do this for both cases, ω_r^{ST} (black dot) and ω_r^{S} (purple dot).

V. AMPLIFICATION

As discussed in the main text, the spatial boundary case presents broadband amplification for certain values of the grating speed, demonstrated by the appearance of horizontal bands of transmission higher than unity in Fig. 3(g). To understand this effect, we study the transmission through a single spatially truncated slab of a STPhC excited by a plane wave of fixed frequency ω_0, while varying the modulation speed c_g. As observed in Fig. S2(a), the transmittance spectrum presents periodic peaks of amplification, whose magnitude increases with c_g (see top x-axis). To understand the periodic nature of the amplification, we also plot the transmittance in terms of the time the wave takes to traverse the slab (see bottom x-axis),

$$t_{\mathrm{slab}} = \frac{D}{v_{\mathrm{eff}}(c_g)}, \tag{87}$$

where D is the size of the slab. When normalized by the modulation period T, we find that the wave is transmitted without amplification when it exits the slab after an integer number of modulation cycles ($t_{\mathrm{slab}} = qT$, with $q \in \mathbb{N}$), and experiences amplification when it exits halfway through the modulation ($t_{\mathrm{slab}} = (q + 1/2)T$). This behaviour can also be interpreted in terms of the permittivity encountered at the slab boundaries. Indeed, for $t_{\mathrm{slab}} = qT$, the wave enters and exits through the same value of the permittivity ($\epsilon^{(\mathrm{in})} = \epsilon^{(\mathrm{out})}$), whereas for $t_{\mathrm{slab}} = (q + 1/2)T$, it encounters opposite permittivities, with $\epsilon^{(\mathrm{in})} = \epsilon_0 \epsilon_m (1 \pm \alpha_e)$ and $\epsilon^{(\mathrm{out})} = \epsilon_0 \epsilon_m (1 \mp \alpha_e)$. Therefore, amplification originates from the boundary conditions themselves, explaining both its broadband character and its independence from the slab thickness.

Finally, using our semi-analytical approach, we analyze the mode decomposition of the reflected, internal and transmitted wave for two representative cases: the modulation is strong but the wave is only transmitted

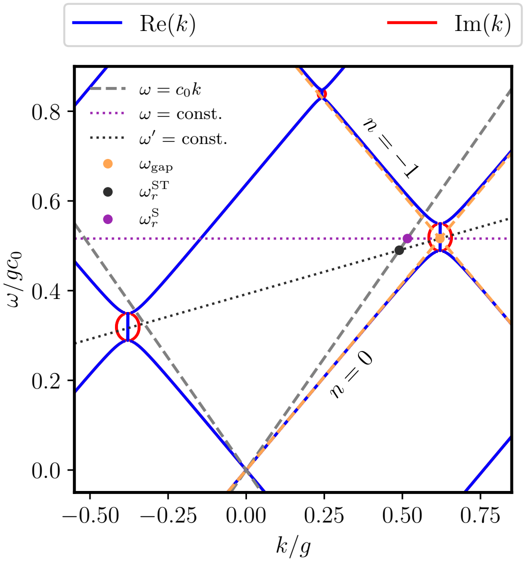

FIG. S1: Representation of the band-crossing position and the resonance frequencies of the interface states for both types of boundaries. The grey and amber dashed lines indicate the free-space dispersion and the linear approximation of the STPhC band dispersion, respectively. The violet and black dotted lines represent the frequency conservation conditions in the lab and comoving frames, respectively. The band-crossing frequency ω_{gap} is obtained from the intersection between the $n = 0$ forward and $n = -1$ backward modes, while the resonance frequencies are determined by applying the corresponding conservation law for each boundary type.

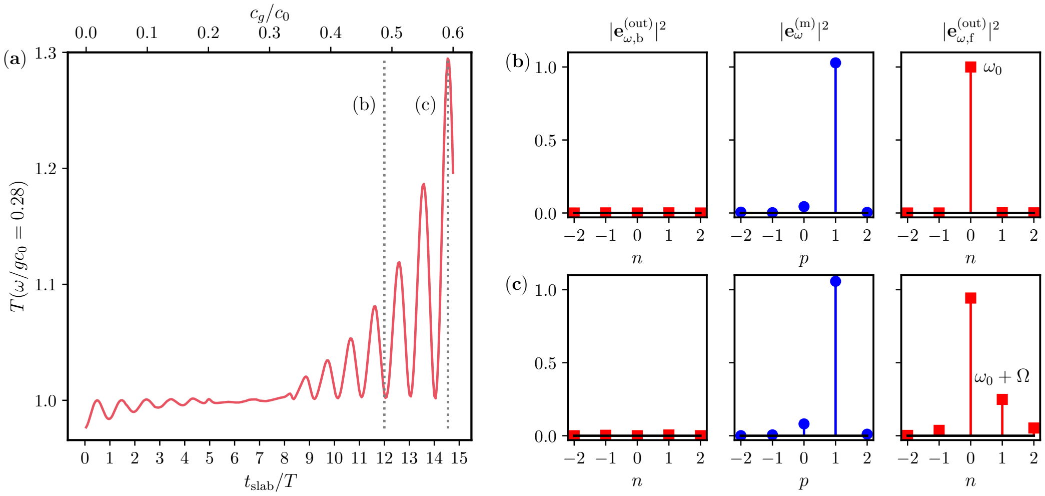

FIG. S2: Amplification of the transmitted wave through higher-frequency mode generation in a spatial slab of a STPhC. (a) Transmittance of a plane wave with $\omega_0 = 0.28gc_0$ exciting a single slab, composed of ten unit cells truncated in space, as a function of the grating velocity c_g and the transit time across the slab t_{slab}, normalized by the modulation period $T = 2\pi/\Omega$. (b) Mode decomposition of the reflected, internal and transmitted wave for a grating velocity corresponding to a unit transmittance. (c) Same magnitudes for a c_g yielding amplification.

and not amplified [Fig. S2(b)], and the maximum amplification point [Fig. S2(c)]. For both cases, reflection is negligible and two internal modes are excited, with the dominant mode ($p = 1$) only containing the fundamental Bloch-Floquet harmonic ($n = 0$), and the $p = 0$ mode including the $n = 1$ harmonic.[1] Thus,

[1] In this section, we omit the τ index for clarity, so that all $2\,(2N_{\text{F}} + 1)$ eigenvalues are labeled just by p. Since the specific value of p has no physical meaning, the eigenvalues can be ordered arbitrarily, and our analysis can focus solely on the Bloch-Floquet harmonics associated with each eigenfunction.

higher-frequency components are present inside the slab in both regimes, though weakly. Importantly, however, the transmitted wave only presents both $n = 0$ and $n = 1$ harmonics in the amplifying case Fig. S2(c), confirming that frequency conversion processes underlie the broadband amplification observed in this system. Indeed, while the weight of each individual harmonic mode cannot exceed unity, the presence of multiple harmonics, combined with the temporal modulation that breaks energy conservation, can result in the sum of their weights being higher than one, thus leading to amplification.

VI. SUPERLUMINAL REGIME

A. Eigenvalue problem for the time-like frame

To study the symmetries of the STPhC in the superluminal regime, we need to make a Lorentz transformation to a frame whose velocity is given by $c_f = c_0^2/c_g$ rather than c_g, coined the time-like frame. In this frame, the magneto-electric coupling is still present and, thus, we derive an eigenvalue problem for ω' starting from the lab-frame ansatz as was done for the subluminal case:

$$\begin{bmatrix} E(x,t) \\ H(x,t) \end{bmatrix} = e^{i(kx-\omega t)} \sum_n \begin{bmatrix} E_n \\ H_n \end{bmatrix} e^{i\,n(gx-\Omega t)} = e^{i(k'x'-\omega' t')} \sum_n \begin{bmatrix} E_n \\ H_n \end{bmatrix} e^{-i\,n\Omega/\gamma\, t'} = \begin{bmatrix} E(x',t') \\ H(x',t') \end{bmatrix}. \quad (88)$$

Then, using Eq. (31) and swapping c_g by c_f, we obtain the following eigenvalue problem:

$$\begin{pmatrix} \mathbb{M}^{\mathrm{EE,L}}(k') & \mathbb{M}^{\mathrm{EH,L}}(k') \\ \mathbb{M}^{\mathrm{HE,L}}(k') & \mathbb{M}^{\mathrm{HH,L}}(k') \end{pmatrix} \begin{bmatrix} \mathbf{E}_{k'} \\ \mathbf{H}_{k'} \end{bmatrix} = \omega'_{k'} \begin{pmatrix} \mathbb{M}^{\mathrm{EE,R}}(k') & \mathbb{M}^{\mathrm{EH,R}}(k') \\ \mathbb{M}^{\mathrm{HE,R}}(k') & \mathbb{M}^{\mathrm{HH,R}}(k') \end{pmatrix} \begin{bmatrix} \mathbf{E}_{k'} \\ \mathbf{H}_{k'} \end{bmatrix}, \quad (89)$$

where the right matrix entries read

$$\mathbb{M}^{\mathrm{EH,R}}_{n,n'} = \mathbb{M}^{\mathrm{HE,R}}_{n,n'} = -c_f/c_0^2\, \delta_{n,n'}, \quad (90)$$

$$\mathbb{M}^{\mathrm{EE,R}}_{n,n'} = -\epsilon_0\epsilon_m[\delta_{n,n'} + \alpha_e(\delta_{n,n'+1} + \delta_{n,n'-1})], \quad (91)$$

$$\mathbb{M}^{\mathrm{HH,R}}_{n,n'} = -\mu_0\mu_m[\delta_{n,n'} + \alpha_m(\delta_{n,n'+1} + \delta_{n,n'-1})], \quad (92)$$

while the left ones are

$$\mathbb{M}^{\mathrm{EH,L}}_{n,n'} = \mathbb{M}^{\mathrm{HE,L}}_{n,n'} = (k' + n\,g/\gamma)\,\delta_{n,n'}, \quad (93)$$

$$\mathbb{M}^{\mathrm{EE,L}}_{n,n'} = \epsilon_0\epsilon_m(c_f k' + n\,\Omega/\gamma)[\delta_{n,n'} + \alpha_e(\delta_{n,n'+1} + \delta_{n,n'-1})], \quad (94)$$

$$\mathbb{M}^{\mathrm{HH,L}}_{n,n'} = \mu_0\mu_m(c_f k' + n\,\Omega/\gamma)[\delta_{n,n'} + \alpha_m(\delta_{n,n'+1} + \delta_{n,n'-1})]. \quad (95)$$

One can show that this eigenvalue problem is equivalent to that of the subluminal case defined in Eq. (49), upon replacing c_g with c_f. This equivalence is expected, since the Lorentz transformations are applied in the same manner, with only the frame velocity being different. Once the eigenproblem is solved, we can then write the eigenfunctions for a given momentum k' as

$$E_{k',\tau,p}(x',t') = e^{i(k'x'-\omega'_{k',\tau,p}t')} \sum_n E_{k',\tau,p,n}\, e^{-i\,n\Omega/\gamma\, t'} \quad (96)$$

and

$$H_{k',\tau,p}(x',t') = e^{i(k'x'-\omega'_{k',\tau,p}t')} \sum_n H_{k',\tau,p,n}\, e^{-i\,n\Omega/\gamma\, t'}. \quad (97)$$

However, in this frame the relevant fields are $[\mathbf{D}', \mathbf{B}']$. Therefore, we need to apply Lorentz transformations:

$$\begin{bmatrix} D'(x',t') \\ B'(x',t') \end{bmatrix} = \tilde{\mathbf{T}}(t') \begin{bmatrix} E(x',t') \\ H(x',t') \end{bmatrix}, \quad (98)$$

with

$$\tilde{\mathbf{T}}(t') = \gamma \begin{pmatrix} \epsilon(t') & c_f/c_0^2 \\ c_f/c_0^2 & \mu(t') \end{pmatrix} \tag{99}$$

the new transformation matrix. Thus, in the time-like frame the eigenfunctions are defined as

$$D'_{k',\tau,p}(x',t') = e^{i(k'x' - \omega'_{k',\tau,p} t')} \sum_n \gamma \left[\epsilon(t')\, E_{k',\tau,p,n} + c_f/c_0^2 H_{k',\tau,p,n}\right] e^{-i\, n\Omega/\gamma\, t'} \tag{100}$$

and

$$B'_{k',\tau,p}(x',t') = e^{i(k'x' - \omega'_{k',\tau,p} t')} \sum_n \gamma \left[\mu(t')\, H_{k',\tau,p,n} + c_f/c_0^2 E_{k',\tau,p,n}\right] e^{-i\, n\Omega/\gamma\, t'}. \tag{101}$$

Finally, we can derive the eigenvalues and eigenvectors in the vacuum by evaluating $\alpha_e = \alpha_m = 0$, as well as $\epsilon_m = \mu_m = 1$, in Eqs. (89). We obtain

$$\omega'^{\pm}_{k'} = -n\Omega/\gamma \pm c_0 k' \tag{102}$$

with $\omega'^{+}_{n} \equiv \omega'_{k',\mathrm{f},n}$ and $\omega'^{-}_{n} \equiv \omega'_{k',\mathrm{b},n}$. The eigenvectors correspond to

$$\begin{bmatrix} E_{k',\mathrm{f},n} \\ H_{k',\mathrm{f},n} \end{bmatrix} = \begin{bmatrix} 1 \\ \frac{-k'}{\mu_0(\omega'_{k',\mathrm{f},n} + n\Omega/\gamma)} \end{bmatrix} = \frac{1}{\sqrt{1+\eta^{-2}}} \begin{bmatrix} 1 \\ -\eta^{-1} \end{bmatrix}; \quad \begin{bmatrix} E_{k',\mathrm{b},n} \\ H_{k',\mathrm{b},n} \end{bmatrix} = \begin{bmatrix} 1 \\ \frac{-k'}{\mu_0(\omega'_{k',\mathrm{b},n} + n\Omega/\gamma)} \end{bmatrix} = \frac{1}{\sqrt{1+\eta^{-2}}} \begin{bmatrix} 1 \\ \eta^{-1} \end{bmatrix}, \tag{103}$$

where $\eta^2 = \mu_0/\epsilon_0$ is the wave impedance in vacuum.

B. Scattering matrix and transmission spectrum

When the modulation enters the superluminal regime, the STPhC acquires a temporal-like nature. Therefore, the two types of boundaries we consider in this regime are: spatiotemporal when the system is truncated along with the modulation, and purely temporal. As in the subluminal regime, each boundary will conserve different magnitudes: k' for the ST interface and k for the temporal one. Consequently, for each case we must select the eigenfunctions of the STPhC that possess a well-defined k or k', such that we use those obtained from Eq. (19) for temporal boundaries and from Eq. (89) for spatiotemporal ones.

1. *Spatiotemporal Boundary*

We start by defining the scattering matrix of two STPhC slabs truncated with ST interfaces. To do so, we now enforce the continuity of the time-like frame D'_z and B'_y fields at each interface, which are easily defined as $t' = 0, N\gamma T, 2N\gamma T$, where N is the number of ST unit cells (defined as $t' \in [0, \gamma T]$) used to construct each slab. Making use of the field decomposition, we can write the matching conditions using a superposition of the eigenvectors as

$$\begin{bmatrix} \mathbf{D}'_{k'} \\ \mathbf{B}'_{k'} \end{bmatrix}^{(\mathrm{m})} = \tilde{\mathcal{M}}'^{(\mathrm{m})}_{k'}\, \mathbf{e}^{(\mathrm{m})}_{k'}; \quad \begin{bmatrix} \mathbf{D}'_{k'} \\ \mathbf{B}'_{k'} \end{bmatrix}^{(\mathrm{in/out})}_{\mathrm{f/b}} = \tilde{\mathcal{M}}'^{(\mathrm{v})}_{k',\mathrm{f/b}}\, \mathbf{e}^{(\mathrm{in/out})}_{k',\mathrm{f/b}}, \tag{104}$$

with

$$\tilde{\mathcal{M}}'^{(\mathrm{m})}_{k'} = \tilde{\mathbf{T}}'^{(\mathrm{m})} \mathcal{M}^{(\mathrm{m})}_{k'}; \quad \tilde{\mathcal{M}}'^{(\mathrm{v})}_{k',\mathrm{f/b}} = \tilde{\mathbf{T}}'^{(\mathrm{v})} \mathcal{M}^{(\mathrm{v})}_{k',\mathrm{f/b}}, \tag{105}$$

where $\mathcal{M}^{(\mathrm{m})}_{k'}$ and $\mathcal{M}^{(\mathrm{v})}_{k',\mathrm{f/b}}$ correspond to the matrices containing the eigenvectors obtained from Eqs. (89) and (19), respectively. Finally, the $\tilde{\mathbf{T}}'^{(\mathrm{v})}$ and $\tilde{\mathbf{T}}'^{(\mathrm{m})}$ matrices correspond to the transformation matrix of Eq. (99) evaluated at the interfaces for the unmodulated and modulated cases, respectively. The periodicity of the modulation ensures that at the boundaries the matrices read

$$\tilde{\mathbf{T}}'^{(\mathrm{v})} = \gamma \begin{pmatrix} \epsilon_0 \mathbb{1} & c_f/c_0^2 \mathbb{1} \\ c_f/c_0^2 \mathbb{1} & \mu_0 \mathbb{1} \end{pmatrix}; \quad \tilde{\mathbf{T}}'^{(\mathrm{m})} = \gamma \begin{pmatrix} \epsilon_0\epsilon_m(1+2\alpha_e)\mathbb{1} & c_f/c_0^2 \mathbb{1} \\ c_f/c_0^2 \mathbb{1} & \mu_0\mu_m(1+2\alpha_m)\mathbb{1} \end{pmatrix}. \tag{106}$$

When considering time-like interfaces, causality needs to be enforced, which consequently forbids the existence of reflected waves in the same space-time region as the incoming wave. To take this into account, we need to change the structure of Eqs. (61)-(63) in order to enforce the correct continuity equations:

- 1st interface:

$$\begin{bmatrix}\mathbf{D}'_{k'}\\ \mathbf{B}'_{k'}\end{bmatrix}_{\mathrm{f}}^{(\mathrm{in}),1}+\begin{bmatrix}\mathbf{D}'_{k'}\\ \mathbf{B}'_{k'}\end{bmatrix}_{\mathrm{b}}^{(\mathrm{in}),1}=\begin{bmatrix}\mathbf{D}'_{k'}\\ \mathbf{B}'_{k'}\end{bmatrix}^{(\mathrm{m}),1}\rightarrow \tilde{\mathcal{M}}'^{(\mathrm{v})}_{k',\mathrm{f}}\,\mathbf{e}^{(\mathrm{in}),1}_{k',\mathrm{f}}+\tilde{\mathcal{M}}'^{(\mathrm{v})}_{k',\mathrm{b}}\,\mathbf{e}^{(\mathrm{in}),1}_{k',\mathrm{b}}=\tilde{\mathcal{M}}'^{(\mathrm{m})}_{k'}\,\mathbf{e}^{(\mathrm{m}),1}_{k'}. \tag{107}$$

- 2nd interface:

$$\begin{bmatrix}\mathbf{D}'_{k'}\\ \mathbf{B}'_{k'}\end{bmatrix}^{(\mathrm{m}),2}=\begin{bmatrix}\mathbf{D}'_{k'}\\ \mathbf{B}'_{k'}\end{bmatrix}^{(\tilde{\mathrm{m}}),2}\rightarrow \tilde{\mathcal{M}}'^{(\mathrm{m})}_{k'}\,\mathbf{e}^{(\mathrm{m}),2}_{k'}=\tilde{\mathcal{M}}'^{(\mathrm{m})}_{k'}\,\mathcal{P}'\,\mathbf{e}^{(\mathrm{m}),1}_{k'}=\tilde{\mathcal{M}}'^{(\tilde{\mathrm{m}})}_{k'}\,\mathbf{e}^{(\tilde{\mathrm{m}}),2}_{k'}. \tag{108}$$

- 3rd interface:

$$\begin{bmatrix}\mathbf{D}'_{k'}\\ \mathbf{B}'_{k'}\end{bmatrix}^{(\tilde{\mathrm{m}}),3}=\begin{bmatrix}\mathbf{D}'_{k'}\\ \mathbf{B}'_{k'}\end{bmatrix}_{\mathrm{f}}^{(\mathrm{out}),3}+\begin{bmatrix}\mathbf{D}'_{k'}\\ \mathbf{B}'_{k'}\end{bmatrix}_{\mathrm{b}}^{(\mathrm{out}),3}\rightarrow \tilde{\mathcal{M}}'^{(\tilde{\mathrm{m}})}_{k'}\,\mathbf{e}^{(\tilde{\mathrm{m}}),3}_{k'}=\tilde{\mathcal{M}}'^{(\tilde{\mathrm{m}})}_{k'}\,\tilde{\mathcal{P}}'\,\mathbf{e}^{(\tilde{\mathrm{m}}),2}_{k'}=\tilde{\mathcal{M}}'^{(\mathrm{v})}_{k',\mathrm{f}}\,\mathbf{e}^{(\mathrm{out}),3}_{k',\mathrm{f}}+\tilde{\mathcal{M}}'^{(\mathrm{v})}_{k',\mathrm{b}}\,\mathbf{e}^{(\mathrm{out}),3}_{k',\mathrm{b}}. \tag{109}$$

$\mathcal{P}'$ corresponds to the diagonal matrix containing the phase acquired by each eigenvector between surfaces $\mathcal{P}'_{jj}=\exp(-i\,\omega'_j D')$, where $D'=N\gamma T$ is the width of each slab and ω'_j the eigenvalues of the STPhC. As we can see, now only incoming waves are allowed before the STPhC slab, while only output waves are present afterwards. Therefore, the scattering matrix connecting input and output waves will have the structure of a transfer matrix. To derive it, we can use the same derivation as in the subluminal case to arrive at a similar expression as Eq. (66):

$$\tilde{\mathbf{N}}_{k'}\tilde{\mathcal{M}}'^{(\mathrm{v})}_{k',\mathrm{f}}\,\mathbf{e}^{(\mathrm{out}),3}_{k',\mathrm{f}}-\tilde{\mathcal{M}}'^{(\mathrm{v})}_{k',\mathrm{b}}\,\mathbf{e}^{(\mathrm{in}),1}_{k',\mathrm{b}}=\tilde{\mathcal{M}}'^{(\mathrm{v})}_{k',\mathrm{f}}\,\mathbf{e}^{(\mathrm{in}),1}_{k',\mathrm{f}}-\tilde{\mathbf{N}}_{k'}\tilde{\mathcal{M}}'^{(\mathrm{v})}_{k',\mathrm{b}}\,\mathbf{e}^{(\mathrm{out}),3}_{k',\mathrm{b}}. \tag{110}$$

Rearranging and writing the equation in vector form we have

$$\underbrace{\tilde{\mathbf{N}}_{k'}\left[\tilde{\mathcal{M}}'^{(\mathrm{v})}_{k',\mathrm{f}},\tilde{\mathcal{M}}'^{(\mathrm{v})}_{k',\mathrm{b}}\right]}_{\tilde{\mathbf{A}}}\begin{bmatrix}\mathbf{e}^{(\mathrm{out}),3}_{k',\mathrm{f}}\\ \mathbf{e}^{(\mathrm{out}),3}_{k',\mathrm{b}}\end{bmatrix}=\underbrace{\tilde{\mathbf{N}}_{k'}\left[\tilde{\mathcal{M}}'^{(\mathrm{v})}_{k',\mathrm{f}},\tilde{\mathcal{M}}'^{(\mathrm{v})}_{k',\mathrm{b}}\right]}_{\tilde{\mathbf{B}}}\begin{bmatrix}\mathbf{e}^{(\mathrm{in}),1}_{k',\mathrm{f}}\\ \mathbf{e}^{(\mathrm{in}),1}_{k',\mathrm{b}}\end{bmatrix}, \tag{111}$$

such that the scattering matrix for the superluminal scattering problem is

$$\begin{bmatrix}\mathbf{e}^{(\mathrm{out}),3}_{k',\mathrm{f}}\\ \mathbf{e}^{(\mathrm{out}),3}_{k',\mathrm{b}}\end{bmatrix}=\tilde{\mathbf{S}}_{k'}\begin{bmatrix}\mathbf{e}^{(\mathrm{in}),1}_{k',\mathrm{f}}\\ \mathbf{e}^{(\mathrm{in}),1}_{k',\mathrm{b}}\end{bmatrix}, \tag{112}$$

with $\tilde{\mathbf{S}}_{k'}=\tilde{\mathbf{A}}^{-1}\,\tilde{\mathbf{B}}$.

Furthermore, we can calculate the fields inside the slabs by writing the weights $\mathbf{e}^{(\mathrm{m}),1}_{k'}$ and $\mathbf{e}^{(\tilde{\mathrm{m}}),2}_{k'}$ in terms of the input weights using Eqs. (107)-(108):

$$\mathbf{e}^{(\mathrm{m}),1}_{k'}=\underbrace{\left(\tilde{\mathcal{M}}'^{(\mathrm{m})}_{k'}\right)^{-1}\left[\tilde{\mathcal{M}}'^{(\mathrm{v})}_{k',\mathrm{f}},\tilde{\mathcal{M}}'^{(\mathrm{v})}_{k',\mathrm{b}}\right]}_{\tilde{\mathbf{C}}}\begin{bmatrix}\mathbf{e}^{(\mathrm{in}),1}_{k',\mathrm{f}}\\ \mathbf{e}^{(\mathrm{in}),1}_{k',\mathrm{b}}\end{bmatrix}, \tag{113}$$

$$\mathbf{e}^{(\tilde{\mathrm{m}}),2}_{k'}=\left(\tilde{\mathcal{M}}'^{(\tilde{\mathrm{m}})}_{k'}\right)^{-1}\tilde{\mathcal{M}}'^{(\mathrm{m})}_{k'}\mathcal{P}'\tilde{\mathbf{C}}\begin{bmatrix}\mathbf{e}^{(\mathrm{in}),1}_{k',\mathrm{f}}\\ \mathbf{e}^{(\mathrm{in}),1}_{k',\mathrm{b}}\end{bmatrix}. \tag{114}$$

Once we have calculated the weights, the fields can be obtained using Eqs. (72)-(73) changing the transformation matrices to the superluminal one defined in Eq. (99), and the exponential term to $e^{ik'x'-i(\omega'_{k',\tau,p}+n\Omega/\gamma)t'}$.

Lastly, the transmission spectrum is defined in the lab-frame by exciting the composite system with a forward propagating plane wave of momentum k_0. As in the subluminal case, we do so by obtaining the transmitted fields and calculating the *space-averaged* Poyting vector, which is defined as

$$P^{(\mathrm{out})}_{k',\mathrm{f}}(t')=-E^{(\mathrm{out})}_{k',\mathrm{f}}(t')\,\left(H^{(\mathrm{out})}_{k',\mathrm{f}}(t')\right)^{*}=-\sum_{n,n'}e^{(\mathrm{out})}_{k',\mathrm{f},n}\,e^{(\mathrm{out})\,*}_{k',\mathrm{f},n'}\,E^{(\mathrm{out})}_{k',\mathrm{f},n}\,H^{(\mathrm{out})\,*}_{k',\mathrm{f},n'}\;e^{-i\left(\omega'_{k',\mathrm{f},n}-\omega'^{*}_{k',\mathrm{f},n'}+(n-n')\Omega/\gamma\right)t'}. \tag{115}$$

Then, normalizing the previous expression by the Poynting vector of the incident wave, and evaluating at the third interface $t' = 2\gamma NT$, we obtain the transmittance as

$$T(k') = \frac{P^{(\mathrm{out})}_{k',\mathrm{f}}(t' = 2\gamma NT)}{P^{(\mathrm{in})}_{k',\mathrm{f}}(t'=0)} = \frac{\sum_{n,n'} e^{(\mathrm{out})}_{k',\mathrm{f},n}\, e^{(\mathrm{out})\,*}_{k',\mathrm{f},n'}\, E^{(\mathrm{out})}_{k',\mathrm{f},n}\, H^{(\mathrm{out})\,*}_{k',\mathrm{f},n'}\; e^{-i\left(\omega'_{k',\mathrm{f},n} - {\omega'}^{*}_{k',\mathrm{f},n'}\right)(2\gamma NT)}}{E^{(\mathrm{in})}_{k',\mathrm{f},0} H^{(\mathrm{in})\,*}_{k',\mathrm{f},0}}. \tag{116}$$

2. Temporal Boundary

Similarly to the subluminal case, the procedure to calculate the scattering matrix for a temporal boundary closely follows the derivation from previous section. However, we need to be mindful of two key differences: the conserved magnitude is now k, and the continuity conditions must be enforced on the lab-frame fields $[D, B]$. To take these differences into account, we need to change the eigenvector matrices of both vacuum and the modulated medium to those defined in Eq. (19) for a given k, as well as the transformation matrix of the STPhC.

In the lab-frame, the transformation matrix $\tilde{\mathbf{T}}^{(m)}$ simply corresponds to the constitutive matrix $\hat{\mathbf{M}}(x,t)$, defined in Eq. (3) from the main text, evaluated at the interfaces. However, evaluating at a temporal interface only gets rid of the temporal dependence, leaving

$$\tilde{\mathbf{T}}^{(m)}(x) = \begin{pmatrix} \epsilon(x) & 0 \\ 0 & \mu(x) \end{pmatrix}. \tag{117}$$

In order to derive the scattering matrix for this composite system, we need to get rid of the spatial dependence too. To do so, we decompose $\epsilon(x)$ and $\mu(x)$ in their Bloch components, which leads to

$$\tilde{\mathbf{T}}^{(m)} = \begin{pmatrix} \mathbf{T}^{\epsilon} & \mathbb{0}_{N_\mathrm{F}\times N_\mathrm{F}} \\ \mathbb{0}_{N_\mathrm{F}\times N_\mathrm{F}} & \mathbf{T}^{\mu} \end{pmatrix}, \tag{118}$$

with

$$\mathbf{T}^{\epsilon}_{n,n'} = \epsilon_0 \epsilon_m [\delta_{n,n'} + \alpha_e(\delta_{n,n'+1} + \delta_{n,n'-1})]; \quad \mathbf{T}^{\mu}_{n,n'} = \mu_0 \mu_m [\delta_{n,n'} + \alpha_m(\delta_{n,n'+1} + \delta_{n,n'-1})]. \tag{119}$$

Finally, we obtain the transmission spectrum by calculating the spatially averaged Poyting vector

$$\begin{aligned} P^{(\mathrm{out})}_{k,\mathrm{f}}(x,t) &= -E^{(\mathrm{out})}_{k,\mathrm{f}}(x,t)\, \left(H^{(\mathrm{out})}_{k,\mathrm{f}}(x,t)\right)^{*} = \\ &= -\sum_{n,n'} e^{(\mathrm{out})}_{k,\mathrm{f},n}\, e^{(\mathrm{out})\,*}_{k,\mathrm{f},n'}\, E^{(\mathrm{out})}_{k,\mathrm{f},n,n}\, H^{(\mathrm{out})\,*}_{k,\mathrm{f},n',n'} e^{i(n-n')gx - i\left(\omega_{k,f,n} - \omega^{*}_{k,f,n'} + (n-n')\Omega\right)t}. \end{aligned} \tag{120}$$

However, similarly to the subluminal case, the fields present an intrinsic spatial dependence due to the nature of the truncation, as we find different values of $\epsilon(x,t)$ and $\mu(x,t)$ along the temporal boundary. We integrate the spatially averaged Poyting vector over one modulation period $\langle P^{(\mathrm{out})}_{k,\mathrm{f}} \rangle = \frac{1}{a}\int_0^a P^{(\mathrm{out})}_{k,\mathrm{f}}$. This allows us to write the transmittance for the temporal boundary case as

$$T(k) = \frac{\langle P^{(\mathrm{out})}_{k,\mathrm{f}}(t = 2NT) \rangle}{\langle P^{(\mathrm{in})}_{k,\mathrm{f}}(t=0) \rangle} = \frac{\sum_n |e^{(\mathrm{out})}_{k,\mathrm{f},n}|^2 E^{(\mathrm{out})}_{k,\mathrm{f},n}\, H^{(\mathrm{out})\,*}_{k,\mathrm{f},n}}{E^{(\mathrm{in})}_{k,\mathrm{f},0} H^{(\mathrm{in})\,*}_{k,\mathrm{f},0}}. \tag{121}$$

C. Spatiotemporal Zak phase

In this section we demonstrate that, as in the subluminal case, the biorthogonal Berry connection is not needed thanks to the Hermiticity of the constitutive matrix and the presence of $\mathcal{P}'\mathcal{T}'$-symmetry. Indeed, starting from Maxwell's equations in the time-like frame for normal incidence and s-polarization

$$\begin{pmatrix} 0 & \partial_{x'} \\ \partial_{x'} & 0 \end{pmatrix} \begin{bmatrix} E'_z \\ H'_y \end{bmatrix} = \partial_{t'} \left(\hat{\mathbf{M}}'(t') \begin{bmatrix} E'_z \\ H'_y \end{bmatrix} \right), \tag{122}$$

we can rearrange them as

$$\partial_{x'} \left(\hat{\mathbf{M}}'(t') \right)^{-1} \begin{bmatrix} D'_z \\ B'_y \end{bmatrix} = \begin{pmatrix} 0 & \partial_{t'} \\ \partial_{t'} & 0 \end{pmatrix} \begin{bmatrix} D'_z \\ B'_y \end{bmatrix}. \tag{123}$$

Then, we can arrive at an eigenvalue problem for the momentum k' by considering $[D'_z, B'_y]$ as plane waves:

$$\hat{\hat{\mathbf{H}}}_{\mathrm{R}}\tilde{\mathbf{\Psi}}'_{\mathrm{R}} = \hat{\mathbf{M}}'(t')\hat{\mathbb{L}}_{t'}\tilde{\mathbf{\Psi}}'_{\mathrm{R}} = k'\tilde{\mathbf{\Psi}}'_{\mathrm{R}}, \;\; \text{with} \;\; \hat{\mathbb{L}}_{t'} = \begin{pmatrix} 0 & -i\partial_{t'} \\ -i\partial_{t'} & 0 \end{pmatrix}, \;\; \text{and} \;\; \tilde{\mathbf{\Psi}}'_{\mathrm{R}} = \begin{bmatrix} D'_z \\ B'_y \end{bmatrix}. \tag{124}$$

Again, the operator $\hat{\hat{\mathbf{H}}}_{\mathrm{R}}$ is not Hermitian because, even though both $\hat{\mathbf{M}}'(t')$ and $\hat{\mathbb{L}}_{t'}$ are, they do not commute. We can obtain its adjoint operator writing

$$\hat{\hat{\mathbf{H}}}_{\mathrm{L}} = \hat{\mathbb{L}}_{t'}\hat{\mathbf{M}}'(t') = \left(\hat{\hat{\mathbf{H}}}_{\mathrm{R}}\right)^{\dagger}, \;\; \text{with} \;\; \hat{\hat{\mathbf{H}}}_{\mathrm{L}}\tilde{\mathbf{\Psi}}'_{\mathrm{L}} = (k')^{*}\tilde{\mathbf{\Psi}}'_{\mathrm{L}}. \tag{125}$$

Similar to the subluminal case, we can rewrite the adjoint operator and its eigenvalue equation as

$$\left(\hat{\mathbf{M}}'(t')\right)^{-1} \underbrace{\hat{\mathbf{M}}'(t')\hat{\mathbb{L}}_{t'}}_{\hat{\hat{\mathbf{H}}}_{\mathrm{R}}} \hat{\mathbf{M}}'(t')\tilde{\mathbf{\Psi}}'_{\mathrm{L}} = (k')^{*}\tilde{\mathbf{\Psi}}'_{\mathrm{L}} \rightarrow \hat{\hat{\mathbf{H}}}_{\mathrm{R}}\hat{\mathbf{M}}'(t')\tilde{\mathbf{\Psi}}'_{\mathrm{L}} = (k')^{*}\,\hat{\mathbf{M}}'(t')\tilde{\mathbf{\Psi}}'_{\mathrm{L}}. \tag{126}$$

Therefore, given that the $\mathcal{P}'\mathcal{T}'$-broken phases are only encountered at the band gaps characterized by complex frequencies ω', we know $\mathcal{P}'\mathcal{T}'$-symmetry will be preserved in the bands, resulting in real k' eigenvalues. In turn, we can write

$$\tilde{\mathbf{\Psi}}'_{\mathrm{R}} = \hat{\mathbf{M}}'(t')\tilde{\mathbf{\Psi}}'_{\mathrm{L}}, \tag{127}$$

such that the inner product of left and right eigenfunctions becomes

$$\langle\tilde{\mathbf{\Psi}}'_{\mathrm{L}}|\tilde{\mathbf{\Psi}}'_{\mathrm{R}}\rangle = \int_{\mathrm{UC}} dt' \left(\tilde{\mathbf{\Psi}}'_{\mathrm{L}}\right)^{\dagger} \tilde{\mathbf{\Psi}}'_{\mathrm{R}} = \int_{\mathrm{UC}} dt' \left(\left(\hat{\mathbf{M}}'(t')\right)^{-1}\tilde{\mathbf{\Psi}}'_{\mathrm{R}}\right)^{\dagger} \tilde{\mathbf{\Psi}}'_{\mathrm{R}} = \int_{\mathrm{UC}} dt' \left(\tilde{\mathbf{\Psi}}'_{\mathrm{R}}\right)^{\dagger} \left(\hat{\mathbf{M}}'(t')\right)^{-1} \tilde{\mathbf{\Psi}}'_{\mathrm{R}}. \tag{128}$$

This relation allows us to write the Zak phase along the time-like bands defined with a biorthogonal Berry connection as

$$\theta_m^{\mathrm{ST}} = i\int_{-\Omega/2\gamma}^{+\Omega/2\gamma} d\omega' \,\langle\tilde{\mathbf{u}}'_{\mathrm{L},\omega',m}|\partial_{\omega'}\tilde{\mathbf{u}}'_{\mathrm{R},\omega',m}\rangle = i\int_{-\Omega/2\gamma}^{+\Omega/2\gamma} d\omega' \,\langle\tilde{\mathbf{u}}'_{\mathrm{R},\omega',m}|\left(\hat{\mathbf{M}}'(t')\right)^{-1}\partial_{\omega'}\tilde{\mathbf{u}}'_{\mathrm{R},\omega',m}\rangle, \tag{129}$$

which ends with the same structure as a conventional Berry connection defined with a weighted scalar product, analogous to the subluminal case.

To obtain the ST Zak phase numerically, we note that the eigenvalue problem in the time-like frame with k' as the eigenvalue can be obtained through Eqs. (32) by changing c_g and c_f, as was previously discussed. Then, the eigenfunctions read

$$D'_{\omega',m}(x',t') = e^{i(k'_{\omega',m}x'-\omega't')}\underbrace{\sum_n \gamma\,\left[\epsilon(t')\,E_{k',m,n} + c_f/c_0^2 H_{k',m,n}\right]e^{-in\Omega/\gamma\,t'}}_{\tilde{u}'^{D}_{\omega',m}(t')} \tag{130}$$

and

$$B'_{\omega',m}(x',t') = e^{i(k'_{\omega',m}x'-\omega't')}\underbrace{\sum_n \gamma\,\left[\mu(t')\,H_{k',m,n} + c_f/c_0^2 E_{k',m,n}\right]e^{-in\Omega/\gamma\,t'}}_{\tilde{u}^{B}_{\omega',m}(t')}, \tag{131}$$

where m corresponds to the band index. With the periodic part of each eigenfunction defined, we can then write the discretized ST Zak phase for superluminal modulation as

$$\theta_m^{\mathrm{ST}} = -\mathrm{Im}\,\log\prod_i \langle\tilde{\mathbf{u}}'_{\omega'_i,m}|\mathbf{u}'_{\omega'_i+1,m}\rangle_{(\hat{\mathbf{M}})^{-1}}, \tag{132}$$

where $\tilde{\mathbf{u}}' = [\tilde{u}'^{D}, \tilde{u}'^{B}]^T$, $\langle\cdot|\cdot\rangle_{(\hat{\mathbf{M}})^{-1}}$ corresponds to the weighted scalar product from Eq. (128), and we enforce the periodic gauge condition $\mathbf{u}'_{\omega'=\Omega/2\gamma,m}(t') = e^{i\Omega/\gamma t'}\mathbf{u}'_{\omega'=-\Omega/2\gamma,m}(t')$.

---

[1] E. Cassedy and A. Oliner, Dispersion relations in time-space periodic media: Part I—Stable interactions, Proc. IEEE **51**, 1342 (1963).

[2] P. A. Huidobro, E. Galiffi, S. Guenneau, R. V. Craster, and J. B. Pendry, Fresnel drag in space–time-modulated metamaterials, Proc. Natl. Acad. Sci. U.S.A. **116**, 24943 (2019).

[3] J. Kong, *Electromagnetic Wave Theory*, A Wiley-Interscience publication (Wiley, 1986).

[4] H. Ding and K. Ding, Non-bloch theory for spatiotemporal photonic crystals assisted by continuum effective medium, Phys. Rev. Res. **6**, 033167 (2024).
